# Association between diet quality and all-cause mortality in a large Dutch cohort

**DOI:** 10.1371/journal.pone.0302905

**Published:** 2024-08-23

**Authors:** Daniel Kirk, A. Mireille Baart, Joseph McLean, Edith J. M. Feskens

**Affiliations:** 1 Division of Human Nutrition and Health, Wageningen University, Wageningen, The Netherlands; 2 Department of Twin Research & Genetic Epidemiology, King’s College London, London, United Kingdom; 3 Androlabs, London, United Kingdom; University of Glasgow, UNITED KINGDOM OF GREAT BRITAIN AND NORTHERN IRELAND

## Abstract

Food-based dietary guidelines are helpful for governments and health agencies to encourage healthy eating at the population level. In order to assess adherence to such guidelines, index scores have been developed, the version in the Netherlands being the Dutch Healthy Diet-index (2015) (DHD2015-index), which reflect adherence to the 2015 Dutch dietary guidelines. Because a higher diet quality, i.e. a higher adherence to the dietary guidelines, is associated with better health outcomes, a higher DHD2015-index score would also mean better outcomes on measures of health, such as all-cause mortality. The present study aimed to elucidate this by investigating the association between DHD2015-index score and mortality in the Dutch population using data from 97 999 participants in the Lifelines cohort study. For the analyses, Cox Proportional Hazards regression was used, whilst accounting for age, sex, physiological measurements, exercise, and biochemical and lifestyle variables. There was a strong negative association between DHD2015-index score and mortality. Hazard ratios for DHD2015-index scores below 60 were approximately 1.2x larger than the mean. Every 10 unit increase in DHD2015-index scores between 60 and 90 led to a 0.1 reduction in hazard ratio, and every 10 unit increase between 90 and the highest DHD2015-index scores led to a reduction in hazard ratios of 0.05. The hazard ratio for the lowest quartile of DHD2015-index scores was 1.14 (95% CI = 1.04–1.26), whereas that for the highest quartile was 0.88 (95% CI = 0.84–0.92). Our results show a clear inverse relationship between DHD2015-index score and all-cause mortality.

## Introduction

Food-based dietary guidelines advise a population on how to eat for the purpose of health preservation [1]. Such guidelines exist in many countries, with the version in the Netherlands being the Dutch dietary guidelines in 2015 [2]. Given the impact of diet on health and the current and growing prevalence of nutrition-related chronic diseases in society [3], it is crucial to measure diet quality. This measure can be used to evaluate dietary interventions aimed at improving diet quality and health status. To this end, the Dutch Healthy Diet 2015-index (DHD2015-index) has been developed and validated [4].

Because of the intimate relationship between nutrition and health, it follows that, if DHD2015-index accurately reflects Dutch dietary guidelines adherence and higher adherence to the guidelines means a healthier diet, then a higher DHD2015-index score would also mean better outcomes on measures of health, such as all-cause mortality. Understanding the relationship between DHD2015-index score and outcomes of health would also be advantageous because it would mean that changes in dietary intake, reflected as a change in DHD2015-index score, following nutrition-based public health interventions could also approximate the subsequential impact on all-cause mortality. However, this is yet to have been thoroughly investigated. Previous studies have been done in either small sample sizes or as part of a comparison with other diet quality assessment measures [5,6], and the latter of these used quality-adjusted life years (QALYs), not all-cause mortality, as its final outcome in a linear regression.

The current study aims to investigate the association between diet quality, as quantified by the DHD2015-index, and all-cause mortality. To assess this, a sample from the Lifelines cohort, a large, multi-generational, prospective cohort study with over 150 000 individuals from the north of the Netherlands, was used. In addition to the primary research question, other covariates hypothesized or known to be relevant to all-cause mortality are also investigated for their relationship with survival probability.

## Methods

### Lifelines dataset

Lifelines is a multi-disciplinary prospective population-based cohort study examining in a unique three-generation design the health and health-related behaviours of 167,729 persons living in the North of the Netherlands [7]. It employs a broad range of investigative procedures in assessing the biomedical, socio-demographic, behavioural, physical and psychological factors which contribute to the health and disease of the general population, with a special focus on multi-morbidity and complex genetics. Participants for the Lifelines cohort study were recruited with the help of general practitioner’s practices across the northern three provinces of The Netherlands (Friesland, Groningen and Drenthe) from 2006 until 2013. Within these practices, all patients in the age range of 25–50 years were invited. Exclusion criteria included having a severe mental or physical illness, limited life expectancy (<5 years), and insufficient knowledge of the Dutch language to complete a Dutch questionnaire. Eligible participants received a first questionnaire and were invited to a Lifelines research facility for a comprehensive health assessment. During this visit, participants were also asked to indicate whether family members would be willing to participate in the study, and in the case of a positive response, family members were invited as well. Children were only allowed to participate if one of their parents was included in the study. In addition to this recruitment strategy, inhabitants of the three northern provinces could also register themselves via the Lifelines website. A more detailed description of the total study population of the Lifelines cohort study can be found elsewhere [8].

The data in the Lifelines database was accessed from 8^th^ June 2022 until 11^th^ November 2023. The data was processed and analyzed via access to the Lifelines Research Workspace, where the data was also stored. All participants signed informed consent upon participation, the research was conducted according to the principles of the Declaration of Helsinki the research code UMCG, and the Lifelines protocol was approved by the UMCG Medical Ethical Committee under number 2007/152.

### Study population for the current study

**Fig 1:**
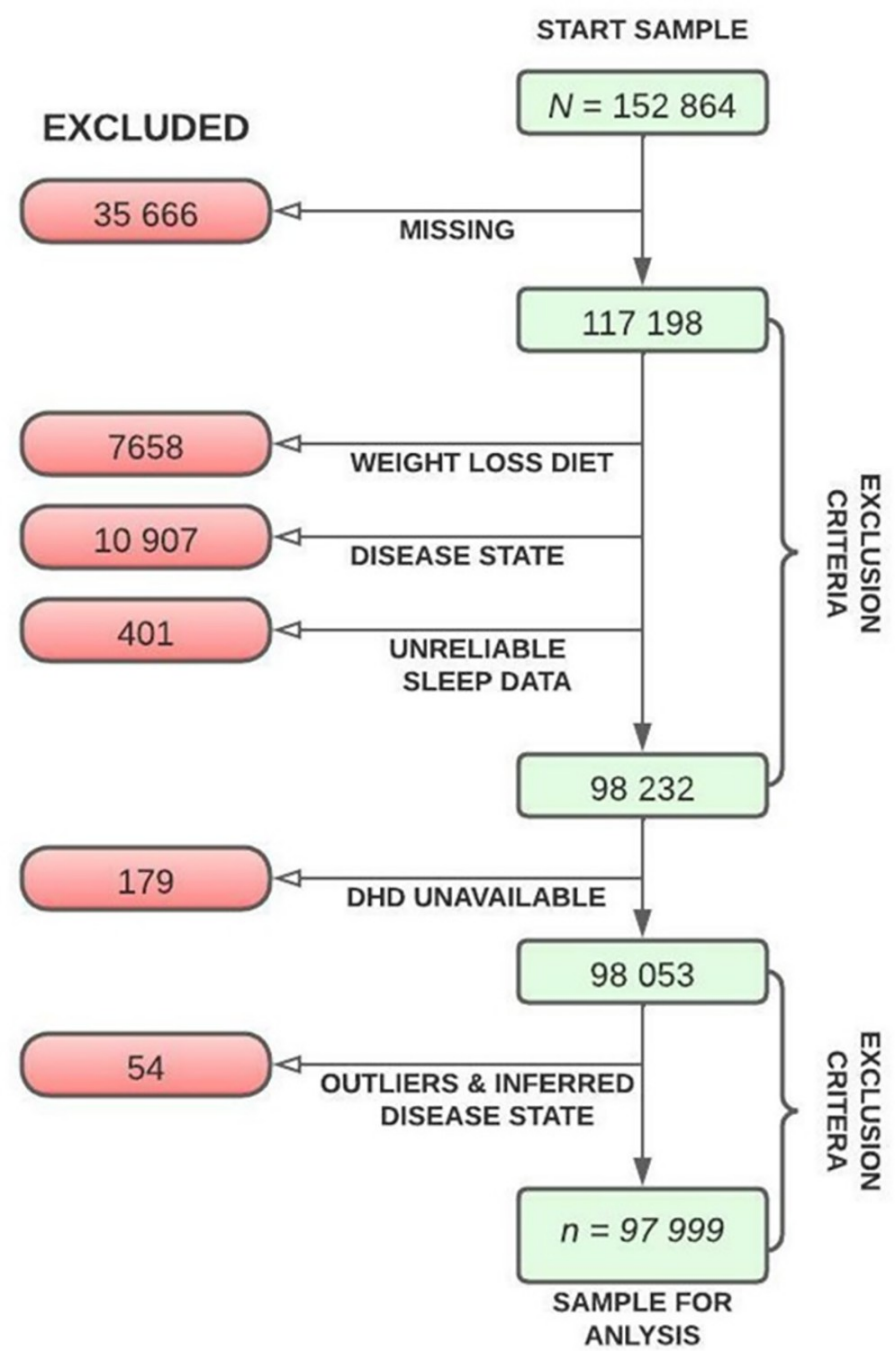
The data processing steps from the starting sample until the final population used in the analysis.

Participants were considered for inclusion for the current study if they were over the age of 18, apparently healthy, and had food frequency questionnaire (FFQ) data available. We excluded all participants who had chronic or serious disease states and for whom the reported dietary intake data may not be reflective of their usual diet. This was achieved primarily on the basis of questionnaire responses but also through examination of outstanding values on boxplots for relevant biological variables (S1 Table in S1 File; S1 Fig in S1 File). Chronic or serious diseases states available on the basis of which participants were excluded were aneurysm or stroke, kidney disease, hepatitis, dementia, Parkinson’s disease, cancer at any point in life, type 2 diabetes, liver cirrhosis, heart attack, heart failure, and having had bypass surgery (N = 10 907). Additionally, participants with outliers in biochemical data which either signified serious or chronic disease or unreliable measurements were also excluded (N = 54; S1 Fig in S1 File, S2 Table in S1 File). Participants who responded “Yes” to the question “are you currently on a diet to lose weight?” (n = 7658) were removed. The number of participants excluded per variable can be seen in Fig 1 with additional information found in S3a and S3b Table in S1 File. The final dataset at the start of the analysis had *N* = 97 999 participants with 1659 events (deaths).

### The Dutch healthy diet 2015-index

Dietary intake data was used to calculate the DHD2015-index [4], which is a measure of adherence to the Dutch dietary guidelines 2015 [2]. The DHD2015-index was derived from the Lifelines data base. A detailed description of the calculation of the DHD2015-index using Lifelines data has been described elsewhere [9].

In short, the DHD2015-index consists of fifteen components: vegetables, fruits, wholegrain products, legumes, nuts, dairy, fish, tea, fats and oils, coffee, red meat, processed meat, sweetened beverages and fruit juices, alcohol, salt and unhealthy choices. Recently, a component on unhealthy foods has been added [10]. The present sixteen components can be divided into adequacy, moderation, optimum, qualitative and ratio components. Adequacy components are derived from a guideline that recommends increasing intake (vegetables, fruits, legumes, nuts, fish, and tea). Moderation components are derived from guidelines that recommend limiting intake (red meat, processed meat, sweetened beverages and fruit juices, alcohol, salt, and unhealthy food choices). Dairy is an optimum component based on an optimal range of intakes, whereas coffee is a qualitative component based on the type of coffee. The fats and oils component is a ratio component and is based on the ratio of intake of healthy and unhealthy products in that food group. The wholegrain products component is considered as two types of components because two guidelines for grain products exist: an adequacy component for wholegrain intake and a ratio component to reflect the replacement of refined grain products by wholegrain products. All components are assigned a score based on intake of the specific food group. For all components, a minimum of 0 points and a maximum of 10 points can be allocated, resulting in a total score ranging from 0 to 160 points, with a higher score indicating better adherence to the guidelines.

Dietary intake in the Lifelines cohort study was assessed using the Flower-FFQ [11,12]. This FFQ consists of one main questionnaire (the heart FFQ) and three complementary questionnaires (the petal FFQs). The heart FFQ contains 110 food items used to estimate intakes of major food groups, energy, and macronutrients. The petal FFQs ask for detailed information on the types of food consumed within the food groups of the heart FFQ, as well as supplement intake, to estimate specific (micro)nutrients and food components. Using data from the Flower-FFQ, the DHD-2015-index was calculated [13]. For the current study, only data from the heart FFQ was used. From the Flower-FFQ, data on filtering of coffee and salt intake is not available, so these two components were not included in the DHD2015-index calculations. Additionally, From the heart-FFQ, regarding the wholegrain products component, only the adequacy component, and not the ratio component, with a maximum of 5 points can be assessed. This results in total scores ranging 0 to 135 points for the DHD2015-index in the current study.

### Description of covariates

We included all variables for which there was a known or potential association with all-cause mortality or eating behavior (S1 Table in S1 File). This included established demographic, lifestyle, and anthropometric variables that have been consistently identified to impact all-cause mortality risk, such as age, BMI, education, smoking status, and physical activity [14–17]. Routinely measured biochemical plasma markers of health, such as lipid and HbA1c levels, among others, were also included [18,19]. Additionally, due to their previous associations with mortality, we included questionnaire responses assessing quality of life [20].

Data on sex, age, smoking, education status, physical activity, and disease status were obtained from questionnaires. Education status was categorized based on education attainment as follows: no education, primary education, lower vocational education, lower general secondary education (low); intermediate vocational education, higher general secondary education (moderate); higher vocational education, and university education (high) [21]. Smoking was categorized as current, former, and never smoker, although current smoker was excluded in favor of Total Number of Cigarettes Smoked per Day. Physical activity was assessed with the short questionnaire to assess health-enhancing physical activity (SQUASH), from which SQUASH moderate and intense activity scores were derived [22]. Anthropometric measurements, including height, weight, and hip and waist circumference, were conducted by well-trained staff at Lifelines research facilities. Biological variables, such as plasma biochemical profile, average blood pressure (BP), and average resting pulse rate were measured as described elsewhere [8]. A collection of quality-of-life variables was also included, which are categorized into questions based on self-rated health on the topics of emotional state, energy, pain state, physical health, social health, general health, and sleep [8].

### Data pre-processing

Survival data were calculated using the difference between date of death and date of enrollment into the study. Participants without a date of death were assumed to be alive and their survival time was calculated as the latest possible date in the dataset subtracted by their enrollment date. All-cause mortality status was also coded accordingly. Only participants over the age of 18 were considered. The starting sample had a size *N* = 152 864. The number of missing data points that were present the variables either to be used in the final model or those used for excluding participants with chronic illness or serious disease can be seen in S3a Table in S1 File. The removal of these missing led to the exclusion of 26 550 participants. No differences in key characteristics were observed before and after the removal of missing data (S3b Table in S1 File). Data reduction was also performed to remove redundant variables (see supplementary section **Data Reduction** S4 Table in S1 File). Data used in the variables included in the model were the baseline data of the participants. Whilst Lifelines has longitudinal information, this information was not used in our analysis. The script for the data processing procedure can be seen in the R file “Data_preprocessing.R”.

### Statistical analysis

The Cox proportion hazards (PH) model was conceived by David Cox to extend life tables, which had been used previously in medical statistics, to incorporate regression-like arguments and allow multivariate analysis of time until an event [23]. The assumptions and the way in which they were tested in the current study are described below. All statistical analyses were done in R version 4.2.2.

### Influential observations

One of the assumptions of the Cox model is that the analysis is free of influential observations, which are data points whose inclusion significantly alters test statistics in a regression model [24]. Influential observations were identified using DFBETAS which quantifies the standard error-scaled change in the estimated regression coefficient upon removal of an observation [25]. DFBETAS plots suggested that 6 six observations from four participants for variables glucose, fasting LDL cholesterol, triglycerides, and HbA1c might have been influential, however since the change in the results before and after the removal of influential observations was insignificant, the data points were ultimately not removed. The interested reader can see the change in the Cox regression summary output in the R code (“Script.R”).

### Linearity

Violation of the linear assumption between predictors and the outcome can lead to erroneous conclusions in the Cox PH model. Typically, Martingale residuals are used to test this assumption, however, this approach is influenced by the smoothing procedure used to produce its plots and is only appropriate when collinearity between the predictors is low, something which cannot be expected from our dataset [26,27]. Thus, instead, we employed a Poisson regression approach as recommended by Therneau [27] for visual assessment of the functional form of continuous, with shapes other than a straight-line suggesting nonlinearity. Penalized splines were used as a formal test for nonlinearity detection [28,29]. Penalized splines add a penalty term to the knots on the curve when minimizing the least squares estimation, which allows the use of more knots and a smoother fit (i.e., avoid overfitting) [30]. Differences in the β coefficients between knots on a spline suggest nonlinearity [28]. Penalized splines are easily implemented by adding ‘pspline’ terms from the Survival package to continuous variables, and evidence of nonlinearity can be quickly gleaned from the output [31].

The results of the Poisson regression and the linear and nonlinear interactions tested by penalized splines can be seen in S2 Fig in S1 File and S5 Table in S1 File, respectively. Taking the results together, it was decided that the variables age, total number of cigarettes smoked per day, hemoglobin, leukocyte, HDL cholesterol, diastolic BP, pulse, systolic BP, and DHD2015-index would be modeled with penalized splines to account for complex relationships with the output variable, whereas linearity would be assumed for the variables BMI, WHR, creatinine, glucose, LDL cholesterol, potassium, triglycerides, and SQUASH moderate and vigorous activity scores.

### Proportional hazards

The assumption that hazards are proportional (i.e., do not change over time) is a crucial characteristic of the Cox model, necessitating that this assumption is verified for each variable in the model and violating offenders are dealt with accordingly [27,28,32]. A formal test of the β coefficient of the Schoenfeld residuals over time shows the statistical significance of the alternative hypothesis that hazards vary with time [33,34], and plots of the Schoenfeld residuals are used to assess if deviation from proportionality is meaningful.

Variables that violate this assumption can be dealt with by adding time-varying coefficients or time-varying covariates [35,36], stratifying by offending categorical variables (or discretization of continuous variables for the same purpose) [27,28], and partitioning of the time-axis [28,37]. It should also be noted that an incorrectly specified functional form for a given variable can cause PH violation, even in another variable [38]. Thus, it should arguably be a first step to experiment with different functional forms, ideally through the implementation of splines, for continuous variables when encountering nonproportionality.

A test on the time-dependency of the coefficient based on the weighted Schoenfeld residuals was done using the function `cox.zph`from the survival package [31] on each occasion that variables were transformed, such as with the addition of splines. This was done over sequential rounds of modelling in order to select an appropriate model whilst maintaining parsimony.

Eventually, penalized splines were added to all continuous variables except Creatine, Glucose and Potassium. The total degrees of freedom used in the model were acceptable given the number of events, and nonproportionality was not deemed problematic in any of the variables of the final model. The proportional hazards test and the Schoenfeld plots for the final model can be seen in S6 Table in S1 File and S3 Fig in S1 File, respectively.

### Final model

The large sample size along with the representativeness of study population for the Dutch general population allowed us to include all of the variables that we considered relevant or potentially relevant for mortality into one model, along with penalized splines to correctly specify the functional form of continuous variables. This led to a final model with 48 variables and 109 degrees of freedom over 1659 events.

## Results

**Fig 2:**
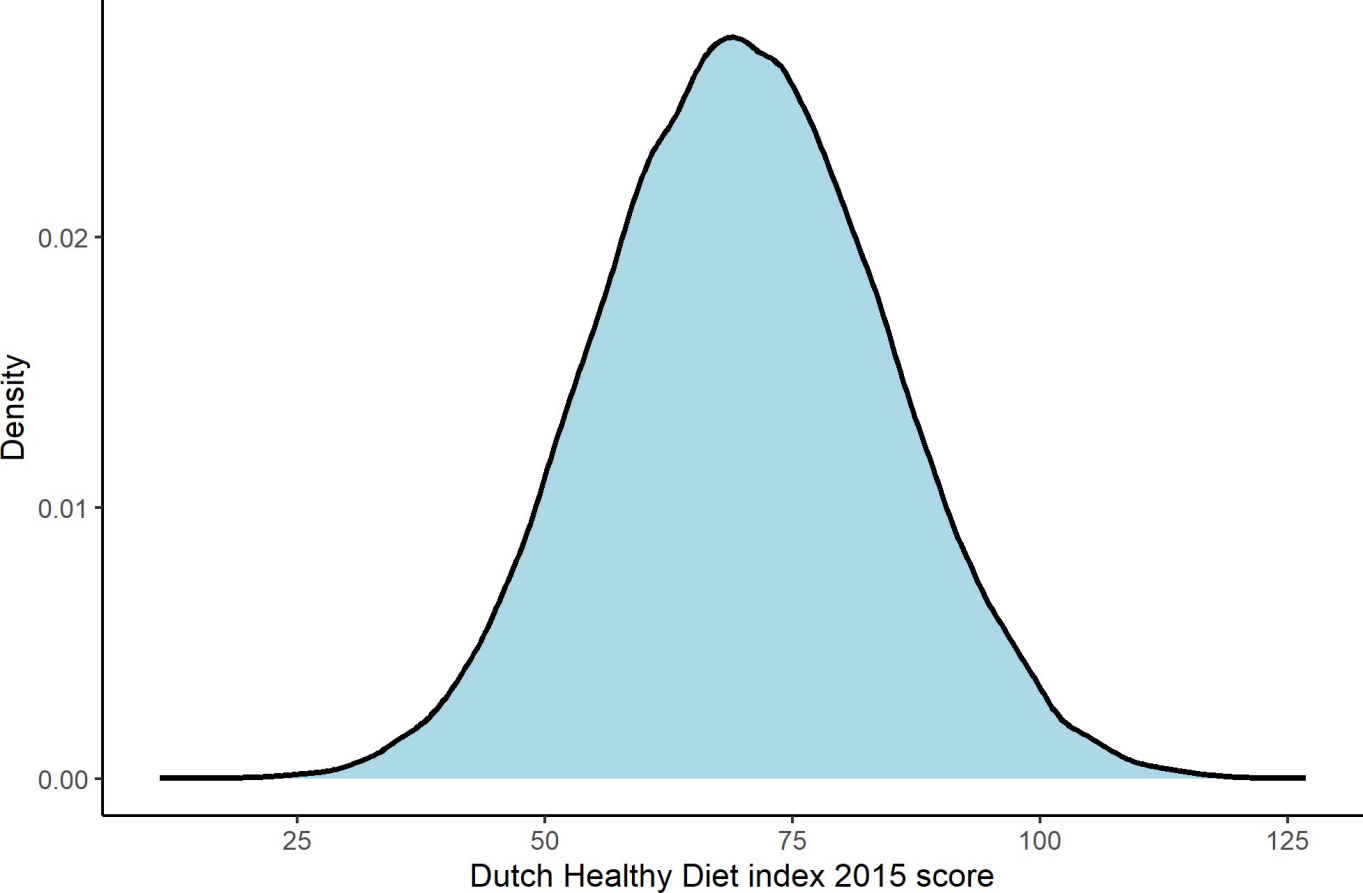
The distribution of DHD2015-index scores in the study population.

**Fig 3:**
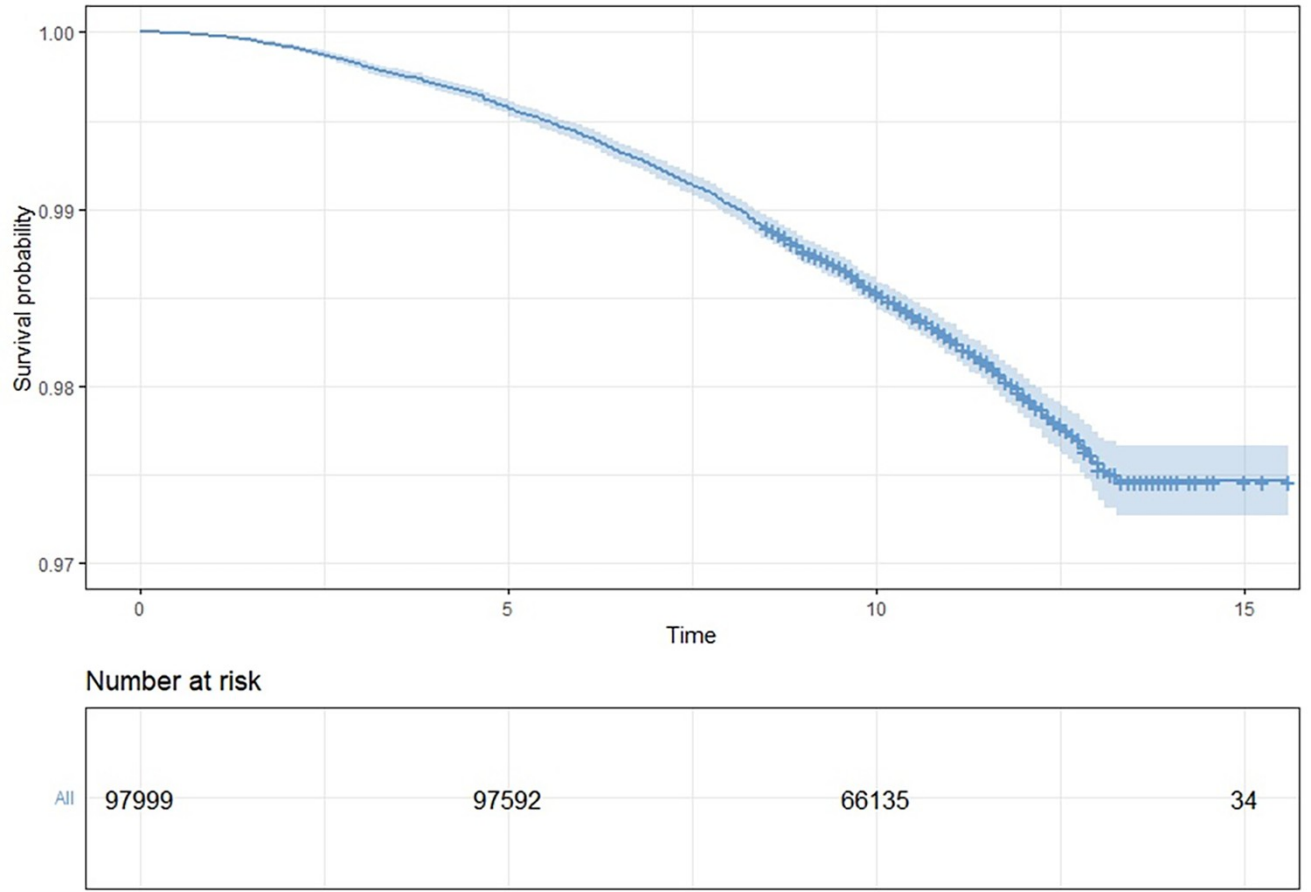
The survival plot of the study population, showing the probability of survival at each time point in years after enrollment into the study. Note that since fewer individuals participated in the study for longer than 10 years, the number at risk is small at longer years of participation.

The main characteristics of the study population after pre-processing can be seen in Table 1. The mean age of the participants was 43.2 years (SD = 12.5, IQR = 34–50) and there were slightly more females (57.0%) than males. Lifelines is a sample of citizens in the north of the Netherlands, and thus most (83.4%) participants identified as being white, with only 1.6% identifying as other, though there was also significant missing data on this aspect (15%). Most participants were moderately or highly educated (70.8%), almost half were never smokers (48.1%), around one-fifth were current smokers (20.7%), and the rest were former smokers (31.1%). Mean energy intake as assessed by food-frequency questionnaire (FFQ) was 2431.4 (SD = 688.8) and 1872.3 (SD = 489) kcal/day in males and females, respectively. Mean BMI was 26.3kg/m^2^ (SD = for males and 25.2 kg/m^2^ for females, however, a larger difference was seen in waist circumference (94.1cm for males and 85.1cm for females. The mean DHD2015-index score was 69.9 (SD = 14.3 overall, 66.9 [SD = 14.1] for males and 72.2 [14.0] for females (Fig 2. Finally, 1.7% (n = 1659) of the population died between the baseline examination and end of follow-up (2.1% in men versus 1.4% women). Fig 3 shows the survival curve. It should be noted that few participants were enrolled for more than 13 years, explaining the abrupt plateau after this timeframe.

**Table 1:** The characteristics of the population sample used in the final model. Continuous variables are given as means (standard deviation).

Characteristics	Total	Male	Female
*Sample Size*	97999	42079	55920
*Age*	43.2 (12.5)	43.69 (12.4)	42.82 (12.6)
*Ethnicity (%)*			
*White/eastern and western European*	83.4	82.2	84.4
*Other*	1.6	1.4	1.7
*NA*	15	16.4	13.9
*Education Level (%)*			
*Low*	26.4	26.8	26.2
*Middle*	40.2	39.2	41.1
*High*	31.6	32.77	30.8
*Mortality Rate (%)*	1.7	2.1	1.4
*Smoking Status (%)*			
*Never*	48.1	45.8	49.9
*Former*	31.1	31.47	31.0
*Current*	20.7	22.73	19.2
*Dutch Healthy Diet Index Score*	69.9 (14.3)	66.9 (14.1)	72.2 (14.0)
*Energy Intake (kcal/day)*	2112 (645)	2431.4 (688.8)	1872.3 (489.0)
*Alcohol Users (%)*	7.6	98.7	97.0
*Alcohol Intake (g/day)*	8.7 (2.7)	8.2 (3.0)	9.1 (2.4)
*BMI (kg/m^2)*	25.6 (4.1)	26.1 (3.5)	25.3 (4.4)
*Waist*:*Hip Ratio*	0.9 (0.08)	1.0 (0.07)	0.9 (0.07)
*Systolic Blood Pressure (mmHg)*	124.6 (14.9)	129.5 (9.4)	120.1 (14.0)
*Diastolic Blood Pressure (mmHg)*	73.4 (9.3)	76.2 (9.4)	71.34 (8.8)
*Hypertension Medication (%)*	7.3	6.92	7.68
*Total Cholesterol (mmol/L)*	5.09 (1.0)	5.17 (1.0)	5.02 (1.0)
*Lipid lowering Medication (%)*	3.7	4.53	3.07
*Fasting Glucose (mmol/L)*	4.9 (0.6)	5.09 (0.7)	4.81 (0.6)
*HbA1c (%)*	5.50 (0.4)	5.52 (0.4)	5.49 (0.4)
*Triglycerides (mmol/L)*	1.15 (0.8)	1.37 (1.0)	0.98 (0.5)

**Fig 4:**
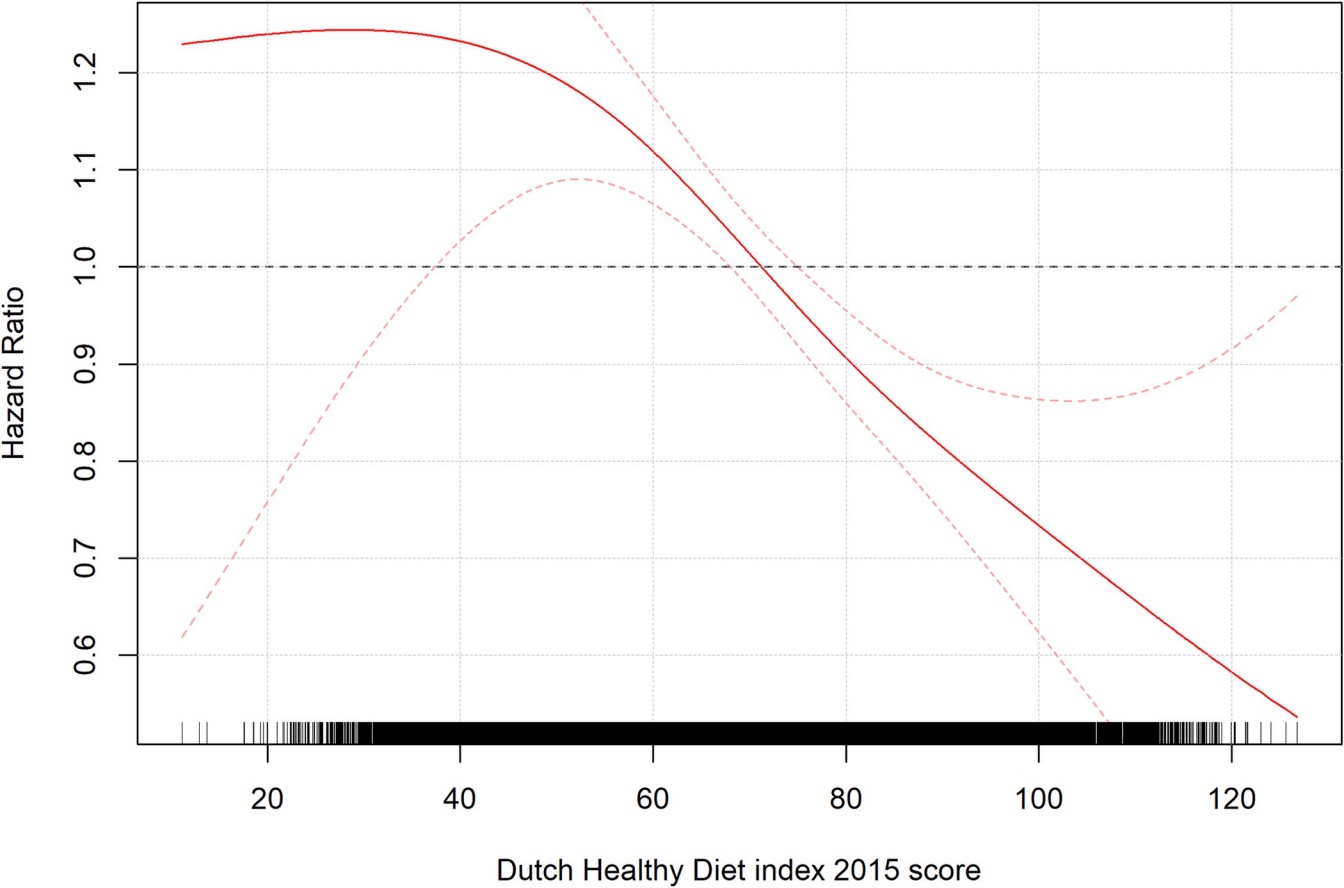
The hazard ratio plot of the DHD2015-index score.

A summary of the results of the Cox regression analyses (final model) is shown in S7 Table in S1 File. DHD2015-index score was strongly associated with all-cause mortality (p<0.001; Table 2). Fig 4. shows how the HR changes over different DHD2015-index scores. The HRs are 1.25 times higher for participants with DHD2015-index below 40 compared to those with the mean DHD2015-index score (69.9), which represented the baseline risk (i.e., HR = 1). An approximately linear decrease in HRs could be seen for scores between 60 and 90, where every 10 unit increase in DHD2015-index score led to a reduction of 0.1 in HR (Fig 4). For participants with scores between 90 and the highest scores (126), every 10 unit increase in DHD2015-index scores led to a reduction in HR of 0.07 (Fig 4). Participants in the lowest quartile had a HR of 1.14 (95% CI = 1.04–1.26) compared with those in the highest quartile, where the HR was 0.88 (95% CI = 0.84–0.92).

**Table 2:** The importance of each variable to the model output, estimated by likelihood ratio tests. An asterisk in the final column represents p<0.05.

Variable	Chi-Squared Statistic	Pr(>|Chi|)	a<0.05
*pspline Age *	1016.843	<0.001	*
*pspline Leukocyte (10^9/L) *	34.33	<0.001	*
*pspline Dutch Healthy Diet Index Score *	24.13	<0.001	*
*pspline HbA1c (%) *	19.576	<0.001	*
*How much of the time feeling tired during the past 4wks*?* *	13.583	0.018	*
*pspline HDL Cholesterol (mmol/L) *	13.04	0.011	*
*Sleep *	11.058	0.086	
*pspline Haemoglobin (mmol/L) *	10.731	0.005	*
*Gender (Female) *	9.561	0.002	*
*pspline LDL Cholesterol (mmol/L) *	8.715	0.068	
*pspline Beats per Minute *	8.578	0.014	*
*pspline Average Diastolic BP (mmHg) *	8.045	0.018	*
*Highest Level of Education *	7.655	0.467	
*I Live Alone (No) *	7.405	0.006	*
*How would you rate your health*, *generally speaking*?* *	7	0.136	
*pspline Average Systolic BP (mmHg) *	6.258	0.044	*
*pspline Total Number Smoked per Day *	5.868	0.052	
*pspline Waist*: *Hip Ratio *	5.154	0.076	
*How much of the time had a lot of energy during the past 4wks*?* *	4.759	0.445	
*pspline Triglycerides (mmol/L) *	4.621	0.099	
*How much of the time been downhearted & blue past 4wks*?* *	4.273	0.51	
*How much of the time been calm & peaceful during the past 4wks*?* *	4.015	0.547	
*Glucose (mmol/L) *	3.656	0.054	
*pspline SQUASH Vigorous Intensity Activity Score *	3.583	0.166	
*pspline SQUASH Moderate Intensity Activity Score *	3.524	0.171	
*My health is excellent *	2.87	0.579	
*How much of the time feeling worn out during the past 4wks*?* *	2.039	0.843	
*Creatinine (mmol/L) *	1.553	0.213	
*Ex-Smoker *	1.165	0.283	
*How much of the time been nervous during the past 4wks*?* *	1.158	0.949	
*Anemia (Yes) *	1.079	0.298	
*I expect my health to get worse *	1.015	0.907	
*Potassium (mmol/L) *	0.955	0.328	
*pspline BMI *	0.881	0.642	
*COPD (No) *	0.744	0.388	
*Hypertension (No) *	0.25	0.616	
*Eating Disorder (Yes) *	0.208	0.647	
*Physical health limited work during past 4wks*?* *	0.138	0.71	
*Current Smoker *	0.122	0.66	
*Gall Stones (Yes) *	0.014	0.907	

Likelihood ratio tests (LRT) were performed in order to estimate the importance of each variable to the model outcome. For each variable, the full model is compared to a model with every variable except one and the ratio of the likelihoods of both the full and reduced model is used to estimate whether the variable significantly contributes to the model [39]. These results are presented in Table 2. Along with DHD2015-index, other important variables in the model were age, number of cigarettes smoked per day, hemoglobin, HbA1c leukocyte levels, HDL cholesterol, diastolic and systolic BP, pulse rate, gender, living alone, and feeling tired during the previous 4 weeks. HRs for all-cause mortality increased as the values for age, number of cigarettes smoked per day, leukocyte and systolic blood pressure levels increased. In contrast, strong negative associations were found for HbA1c and hemoglobin. Variables with U-shaped relationships to mortality included HDL cholesterol, diastolic blood pressure, and pulse rate. Since these variables were modelled with splines and a simple interpretation of their HRs is not available, plots of these hazard ratios can be seen in S4 Fig in S1 File. For the categorical variables, females had a HR of 0.74 (95% CI = 0.61–0.89) compared to males, living alone had a HR of 0.80 (95% CI = 0.69–0.94) compared to not living alone, and “Never”, "Sometimes” or “Rarely” feeling tired in the previous 4 weeks was associated with a higher HR than those feeling tired less often, with those reporting feeling tired “All of the time”, “Most of the time” or “Often” having the lowest HRs (S4 Fig in S1 File).

## Discussion

In a sample of almost 100 000 individuals from the north of the Netherlands, a strong inverse relationship was found between DHD2015-index scores and all-cause mortality [4]. HRs were approximately 1.2x higher than the mean for all DHD2015-index scores below 60 (Fig 4). suggesting that the effects of poor diet as measured by the DHD2015-index on mortality are comparable until this threshold. However, those with the lowest DHD2015-index scores tended to be younger and therefore had a lower risk of death since age was the most influential predictor of all-cause mortality, which may somewhat mask the true harmful effects expected from a low DHD2015-index score. The HR plot of DHD2015-index score was then characterized by two approximately linear lines between 60 and 90, where HR fell from ~1.1 to ~0.81 (a decrease in HR of 0.01 per 1 unit increase in DHD2015-index score), and between 90 and the highest DHD2015-index scores, where HR decreased from ~0.81 to ~0.54 (a decrease in HR of 0.007 per 1 unit increase in DHD2015-index score, This would suggest slightly diminishing returns once DHD2015-index scores reach around 90. Nonetheless, these results suggest a strong relationship whereby all-cause mortality is significantly reduced as DHD2015-index score increases.

The health benefits of key dietary components of the DHD2015-index are well established. High fruit and vegetable consumption consistently correlates with a reduced risk of cardiovascular disease and all-cause mortality [40]. These food groups serve as a rich source of essential micronutrients like vitamins A, C, and E, alongside other health-promoting nutrients like fibre and phytochemicals. The mechanisms underlying these beneficial effects are multifaceted. Dietary fibre and vegetable intake lowers low-density lipoprotein (LDL) cholesterol [41] and blood pressure [42], both established biomarkers for cardiovascular disease [43,44]. Furthermore, dietary fibre intake has also been linked to improved vascular [42] and immune function [45] Additionally, the antioxidants in fruits and vegetables may act as scavengers of reactive oxygen species (ROS), thereby mitigating cellular oxidation and lowering oxidative stress [46]. High consumption levels of these foods (vegetable and fruit) in addition to wholegrains and legumes are well-known dietary intervention targets for weight loss and maintenance [47,48], a separate, well-documented causal variable for cardiovascular disease [49], type 2 diabetes [50] and all-cause mortality [49].

After age, which had the largest effect on all-cause mortality, number of cigarettes smoked per day, gender, systolic and diastolic BP, and heart rate were also significantly related to all-cause mortality, as reported elsewhere [51–58]. Important plasma predictors were plasma leukocyte concentration, which had the highest Chi-Square of the other variables after age on the LRT, hemoglobin, and HDL cholesterol, all of which have established relationships with all-cause mortality [59–70]. Important self-reported variables included living with others (HR = 0.80) and “How much time feeling tired during the past 4 weeks”. Loneliness has been suggested to negatively influence metabolic health and is associated with poorer lifestyle habits [71–73]. Somewhat counterintuitively, “Never” feeling tired in the past 4 weeks had the highest HR, with “Always” having the lowest. The most likely explanation for this is that these levels were also associated with age, with the level “Never” having the oldest average age before decreasing at each level until “Always”, which had the youngest average age (S5 Fig in S1 File). This most likely reveals differences in how younger versus older people report their subjective tiredness, similar to how older people, despite generally having more health problems, more positively evaluate their own health compared to younger people [74].

## Limitations

Some important limitations of the present study should also be noted. Pairwise differences were hardly present in the categorical variables, a reason for which could be the variability of results depending on the selection of the baseline factor. The reference is selected automatically by the R program and is the first level of the factor by default, however, this can be problematic in our case since participants may avoid entering extreme results on categorical data collected using a Likert scale with a middle point [75–78]. Thus, it is possible that the statistical significance of some of the factor levels would be different had another baseline reference been selected. This was not investigated thoroughly since this was not our primary goal, however, no meaningful findings were seen when the factor level was changed for some variables.

It has been argued that the use of splines can lead to overfitting of the data, which may limit the generalizability of our results [29]. Firstly, the Lifelines dataset is considered nationally representative of the Dutch population [79], and our final dataset was large with a sample size of almost 100 000. Despite this, we took steps to limit the possibility of overfitting, including limiting the degrees of freedom with respect to the number of events in the model [28,80,81], limiting the degrees of freedom on splined variables and assuming linearity for certain variables where nonlinearity was insignificant or not meaningful (see supplementary materials for details on the modeling procedure). However, whilst overfitting the data is undesirable, so too is underfitting; given that splines do not make assumptions about the nature of the data, they represent an appropriate way to model complex relationships to a high degree of accuracy, and thus provide a more accurate model. Finally, whilst Cox regression is a well-established approach for answering our research question, our approach was not compared against other alternatives such as modern machine learning techniques [82].

## Conclusion

Adherence to the Dutch dietary guidelines as reflected by the DHD2015-index, is a strong independent predictor of all-cause mortality in the healthy Dutch population. Future work could investigate whether public health nutritional interventions that lead to an increase in diet quality, reflected as an increase in DHD2015-index scores, also reduce mortality.

## Supporting information

S1 File Supporting information for the manuscript “association between diet quality and all-cause mortality in a large Dutch cohort”. (DOCX)

## Abbreviations

BP: blood pressure
DHD2015-index: Dutch healthy diet index 2015
HR: hazard ratio
LRT: likelihood ratio test
