# Supplementary material for: Association between diet quality and all-cause mortality in a large Dutch cohort
Source: PLoS One. 2024 Aug 23;19(8):e0302905. doi: 10.1371/journal.pone.0302905 (PMC11343414; doi:10.1371/journal.pone.0302905)
Supplement: S1 File — (DOCX) [file pone.0302905.s001.docx]

**Supplementary material for: Adherence to the Dutch Healthy Diet Index Lowers All-cause mortality in the Healthy Dutch Population, Kirk et al.**

**Description of Lifeline variables**

Table S1: Each variable selected for the starting analysis after data pre-processing. Some variable names were modified slightly from the name in the Lifelines database in the current paper for interpretability and are given in the first column.

| **Theme** | **Given Variable Name** | **Lifelines Description** |
| --- | --- | --- |
| General & Demographic | Age | Age of participant at baseline |
|  | Gender | Gender of participant (male or female) |
|  | BMI | N/A (calculated using combinations of other variables) |
|  | WHR | N/A (calculated using combinations of other variables) |
|  | Current Smoker | Current smoker (yes or no) |
|  | Ex-Smoker | Ex-Smoker (yes or no) |
|  | Total Number of Cigarettes Smoked per Day | total number smoked per day (all types except e-cigarettes) |
|  | Highest Level of Education | what is the highest level of education you have attained? |
| Biochemical Profile | HbA1c | hba1c in k2-edta tube (%) |
|  | Hemoglobin | hemoglobin in k2-edta tube (mmol/l) |
|  | Leukocyte concentration | leukocytes in k2-edta tube (10e9/l) |
|  | Cholesterol Total | cholesterol total in lithium heparin tube (mmol/l) |
|  | HDL Cholesterol | hdl cholesterol in lithium heparin tube (mmol/l) |
|  | LDL Cholesterol | ldl cholesterol in lithium heparin tube (mmol/l) |
|  | Creatinine | creatinine in lithium heparin tube (umol/l) |
|  | Fasting Glucose | glucose in naf tube (mmol/l) |
|  | Triglycerides | triglycerides in lithium heparin tube (mmol/l) |
|  | Potassium | potassium in lithium heparin tube (mmol/l) |
| Physiological Measurements | Average Arterial Pressure | mean average pressure (mmhg), average over measurements according to [bpavg_calculation_all_m_v1/v2] |
|  | Diastolic BP | diastolic blood pressure (mmhg), average over measurements according to [bpavg_calculation_all_m_v1/v2] |
|  | Beats per Minute | pulse rate in beats per minute, average over measurements according to [bpavg_calculation_all_m_v1/v2] |
|  | Systolic BP | systolic blood pressure (mmhg), average over measurements according to [bpavg_calculation_all_m_v1/v2] |
| Physical Activity | SQUASH Moderate Intensity Score | moderate intensity, activity score (version 2) |
|  | SQUASH Vigorous Intensity Score | vigorous intensity, activity score (version 2) |
| Health Status | Anemia Presence | anemia / could you indicate which of the following disorders you have (had)? |
|  | COPD Presence | do you have copd, emphysema or chronic bronchitis? |
|  | Eating Disorder Presence | eating disorder / could you indicate which of the following disorders you have (had)? |
|  | Gallstones Presence | gallstones / could you indicate which of the following disorders you have (had)? |
|  | Hypertension Presence | have you ever had hypertension? |
|  | I Live Alone | i live alone / which other people are living in your house? (more than half the time) |
|  | Periodontitis Diagnosis | have you been diagnosed with periodontitis by your/a dentist? |
| Quality of Life | How much time nervous during the last 4 weeks? | have you been a very nervous person? / how much of the time during the past 4 weeks |
|  | How much time have you felt calm and peaceful during the last 4 weeks? | have you felt calm and peaceful? / how much of the time during the past 4 weeks |
|  | How much time have you felt downhearted and blue during the last 4 weeks? | have you felt downhearted and blue? / how much of the time during the past 4 weeks |
|  | How much time have you felt happy during the last 4 weeks? | have you been a happy person? / how much of the time during the past 4 weeks |
|  | How much time did you have a lot of energy during the last 4 weeks? | did you have a lot of energy? / how much of the time during the past 4 weeks |
|  | How much time did you feel worn out during the last 4 weeks? | did you feel worn out? / how much of the time during the past 4 weeks |
|  | How much time feeling tired during the last 4 weeks? | did you feel tired? / how much of the time during the past 4 weeks |
|  | How would you rate your health, generally speaking? | how would you rate your health, generally speaking? |
|  | Compared to a year ago, how would you rate your health? | compared to a year ago, how would you rate your health, generally speaking? |
|  | How much has your physical health limited work in the past 4 weeks? | were limited in the kind of work or other activities / during the past 4 weeks, have you had any of the following problems with your work or other regular daily activities as a result of your physical health? |
|  | How much bodily pain have you had during the past 4 weeks? | how much bodily pain have you had during the past 4 weeks? |
|  | How much did pain interfere with normal work during the past 4 weeks? | during the past 4 weeks, how much did pain interfere with your normal work (including both work outside the home and housework)? |
|  | I seem to get sick a little easier than other people | i seem to get sick a little easier than other people |
|  | I expect my health to get worse | i expect my health to get worse |
|  | My health is excellent | my health is excellent |
|  | Does your health limit vigorous activities? | vigorous activities, such as running, lifting heavy objects, participating in strenuous sports / does your health now limit you in the following activities? |
|  | Does your health limit moderate activities? | moderate activities, such as moving a table, pushing a vacuum cleaner, bicycling / does your health now limit you in the following activities? |
|  | Does your health limit climbing one flight of stairs? | climbing one flight of stairs / does your health now limit you in the following activities? |
|  | Does your health limit walking half a kilometer? | walking half a kilometer / does your health now limit you in the following activities? |
|  | To what extent has your physical health or emotional problems interfered with your normal social activities during the past 4 weeks | during the past 4 weeks, to what extent has your physical health or emotional problems interfered with your normal social activities with family, friends, neighbors, or groups? |
|  | How much of the time has your physical health or emotional problems interfered with your social activities during the past 4 weeks? | during the past 4 weeks, how much of the time has your physical health or emotional problems interfered with your social activities (like visiting with friends, relatives, etc.)? |

**Outliers in biochemical variables**

Table S2: Variables that had cut points leading to the exclusion of variables, cut point values (second column, in the units of the variable in the first column) the number of participants excluded after the application of each cut point, and references supporting cut points.

| **Variable** | **Cut-off Point** | ***n* Excluded** | **References** |
| --- | --- | --- | --- |
| *Leukocyte (10^9^/L)* | ≥20 | 14 | (Blumenreich 1990; Ernst et al. 1987) |
| *Creatinine (umol/L)* | ≥100 | 9 | (Burtis, Ashwood, and Bruns 2011) |
| *Potassium (mmol/L)* | ≥6 | 4 | (Burtis et al. 2011) |
| *Hemoglobin (mmol/L)* | ≥12 | 1 | (Vieth and Lane 2014) |
| *Average Arterial Blood Pressure (mmHg)* | >0 | 1 | N/A |
| *Resting heart rate (Beats Per Minute)_* | ≥135 | 4 | (Quer et al. 2020) |
| *Triglycerides (mmol/L)* | ≥18 | 5 | (Feather et al. 2020) |
| *Total Cholesterol (mmol/L)* | ≥12 | 6 | (Lee and Siddiqui 2021) |
| *Glucose (mmol/L)* | ≥20 | 9 | (Lee and Siddiqui 2021) |

**Missing Data**

Table S3a: Characteristics of the data before and after removing missing values.

| **Characteristic** | **Final Dataset** | **Start Sample** |
| --- | --- | --- |
| *Size* | 97999 | 152864 |
| *Age* | 43.2 | 44.4 |
| *Female (%)* | 55.13 | 57.06 |
| *White (%)* | 83.41 | 78.19 |
| *Smoking Status* |  |  |
| *Never* | 48.1 | 42.9 |
| *Ever* | 31.1 | 30.60 |
| *Current* | 20.7 | 19.91 |
| *Mean DHD Index Score* | 69.9 | 69.8 |
| *Mean BMI* | 25.6 | 26.0 |

Table S3b: The number of missing values from each variable:

| **Variable** | **Number Missing** |
| --- | --- |
| bodyweight_diet_adu_q_1 | 753 |
| squashsum_activityscore_adu_c_2_b | 0 |
| squashsum_activityscore_adu_c_2_c | 0 |
| age | 1161 |
| gender | 1161 |
| anemia_presence_adu_q_1 | 1161 |
| aneurysm_diagnosis_adu_q_1 | 1556 |
| angioplasty_bypass_adu_q_1 | 1775 |
| cancer_lifetime_adu_q_1 | 1292 |
| copd_presence_adu_q_1 | 1766 |
| degree_highest_adu_q_1 | 1564 |
| dementia_presence_adu_q_1 | 14485 |
| diabetes_type_adu_q_1 | 1161 |
| eatingdisorder_presence_adu_q_1 | 1161 |
| gallstones_presence_adu_q_1 | 1161 |
| heartattack_presence_adu_q_1 | 1582 |
| heartfailure_presence_adu_q_1 | 2838 |
| hepatitis_presence_adu_q_1 | 1161 |
| hypertension_presence_adu_q_1 | 1220 |
| inhouse_alone_adu_q_1 | 2899 |
| kidneydisease_diagnosis_adu_q_1 | 6964 |
| livercirrhosis_presence_adu_q_1 | 1161 |
| parkinsons_presence_adu_q_1 | 14485 |
| periodontitis_diagnosis_adu_q_1 | 14957 |
| rand_emotional_adu_q_09_b | 1212 |
| rand_emotional_adu_q_09_d | 1281 |
| rand_emotional_adu_q_09_f | 1268 |
| rand_emotional_adu_q_09_h | 1315 |
| rand_energy_adu_q_09_e | 1375 |
| rand_energy_adu_q_09_g | 1392 |
| rand_energy_adu_q_09_i | 1225 |
| rand_generalhealth_adu_q_01 | 1183 |
| rand_generalhealth_adu_q_02 | 1193 |
| rand_limitations_adu_q_04_c | 1319 |
| rand_pain_adu_q_07 | 1294 |
| rand_pain_adu_q_08 | 1351 |
| rand_perception_adu_q_11_a | 1222 |
| rand_perception_adu_q_11_c | 1265 |
| rand_perception_adu_q_11_d | 1236 |
| rand_physical_adu_q_03_a | 1286 |
| rand_physical_adu_q_03_b | 1226 |
| rand_physical_adu_q_03_e | 1427 |
| rand_physical_adu_q_03_h | 1268 |
| rand_social_adu_q_06 | 1304 |
| rand_social_adu_q_10 | 2478 |
| sleeping_timespending_adu_q_1 | 14738 |
| sleeping_timespending_adu_q_1_a | 14485 |
| stroke_presence_adu_q_1 | 1863 |
| hba1cperc_result_all_m_1 | 1544 |
| hemoglobin_result_all_m_1 | 1035 |
| leukocyte_result_all_m_1 | 1034 |
| cholesterol_result_all_m_1 | 780 |
| creatinine_result_all_m_1 | 779 |
| glucose_result_all_m_1 | 1328 |
| hdlchol_result_all_m_1 | 780 |
| ldlchol_result_all_m_1 | 789 |
| potassium_result_all_m_1 | 790 |
| triglyceride_result_all_m_1 | 780 |
| bodylength_cm_all_m_1 | 115 |
| bodyweight_kg_all_m_1 | 72 |
| circumference_hip_all_m_1 | 116 |
| circumference_waist_all_m_1 | 72 |
| bpavg_arterial_all_m_1 | 143 |
| bpavg_diastolic_all_m_1 | 135 |
| bpavg_pulse_all_m_1 | 152 |
| bpavg_systolic_all_m_1 | 135 |
| current_smoker_adu_c_2 | 203 |
| ex_smoker_adu_c_2 | 203 |
| total_frequency_adu_c_1 | 9295 |

**Data Reduction**

Data reduction was done to save on degrees of freedom and remove redundant variables. Covariates for which a possible link to the outcome of interest was hypothesized to be too tenuous or for which the data may be unreliable, such as too large a risk of reverse causality, were removed. This included “How much bodily pain have you had during the past 4 weeks?”, “During the past 4 weeks, how much did pain interfere with your normal work?”, “To what extent has your physical health or emotional problems interfered with your normal social activities during the past 4 weeks” and “How much of the time has your physical health or emotional problems interfered with your social activities during the past 4 weeks?”. Redundancy analysis (redun) from the Hmisc R package (version 4.7-0) was also performed to identify superfluous variables^1^. The “redun” function employs parametric additive models to determine how well each variable can be predicted by the remaining predictor variables (excluding the dependent variable), using R^2^ to score predictive capability^1^. An R^2^ ¬¬cutoff of 0.90 was chosen to determine redundant variables, which resulted in the removal of the variables “Cholesterol Total” and “Average Arterial Pressure”.

Table S3: Significant changes in covariates in Cox regression results following the removal of redundant variables.

| **Variable** | **Coefficient [CI]** | **Significance (p-value)** | **Robust Standard Error** | **Coefficient [CI]** | **Significance (p-value)** | **Robust Standard Error** |
| --- | --- | --- | --- | --- | --- | --- |
|  | Before Redundancy Analysis | | | After Redundancy Analysis | | |
| LDL Cholesterol | 0.806 [0.607-1.070] | 0.137 | 0.145 | 0.910 [0.859-0.964] | 0.001 | 0.0029 |
| Systolic Blood Pressure | 1.000 [0.988-1.012] | 0.962 | 0.00613 | 1.007 [1.003-1.010] | 0.001 | 0.0021 |

**Exclusion of Unhealthy**

Based on a combination of reference ranges identified in the literature and visual assessment of boxplots (Figure S1), plausible cut-points by which to exclude participants were those seen in eTable3 (note that the number of participants excluded after each cut-point application is sensitive to the order in which the variables were processed). Finally, sleep data came as two separate variables, sleep in hours and sleep in minutes; these were converted simply to hours and then rounded to the nearest hour.
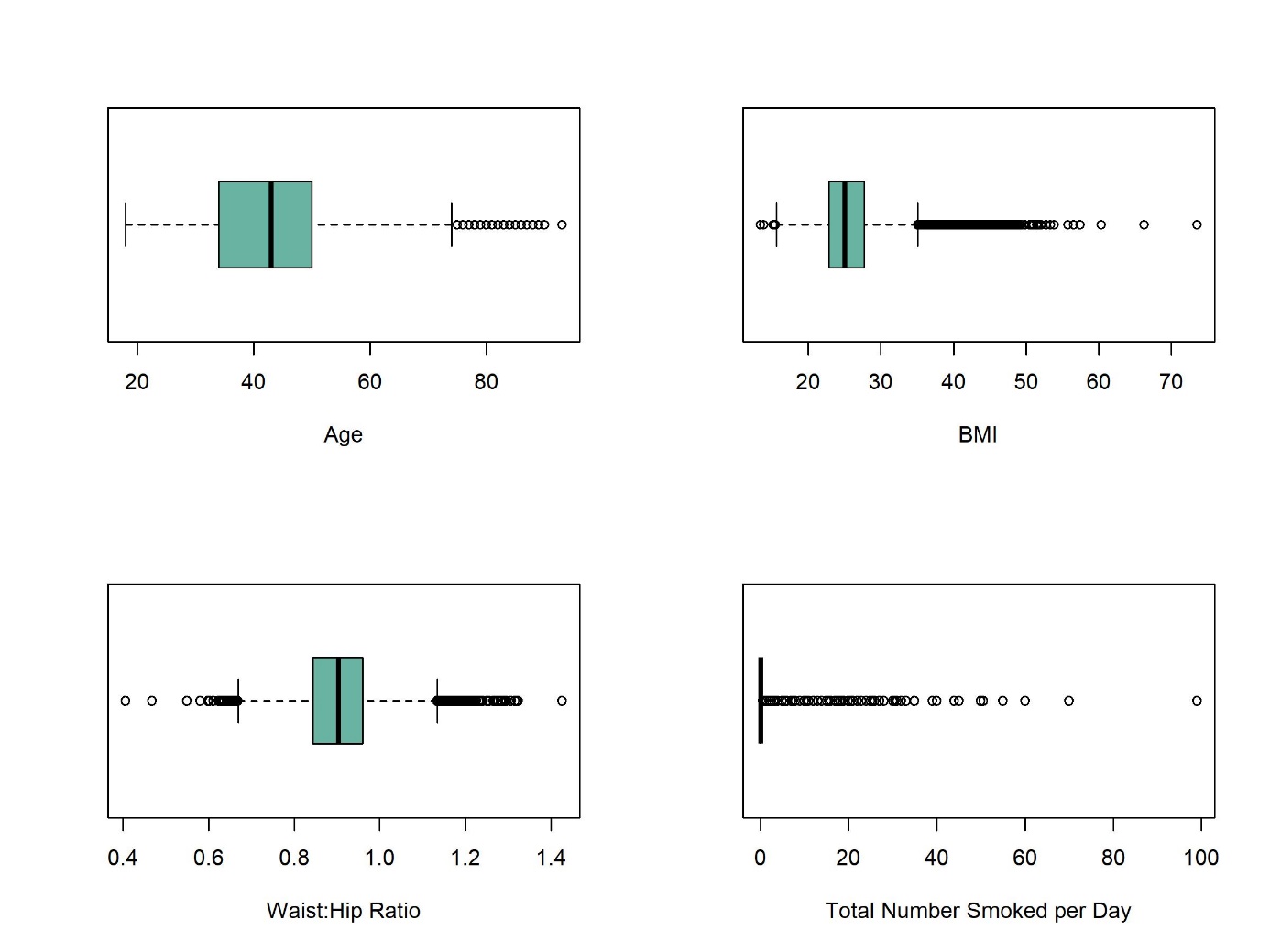

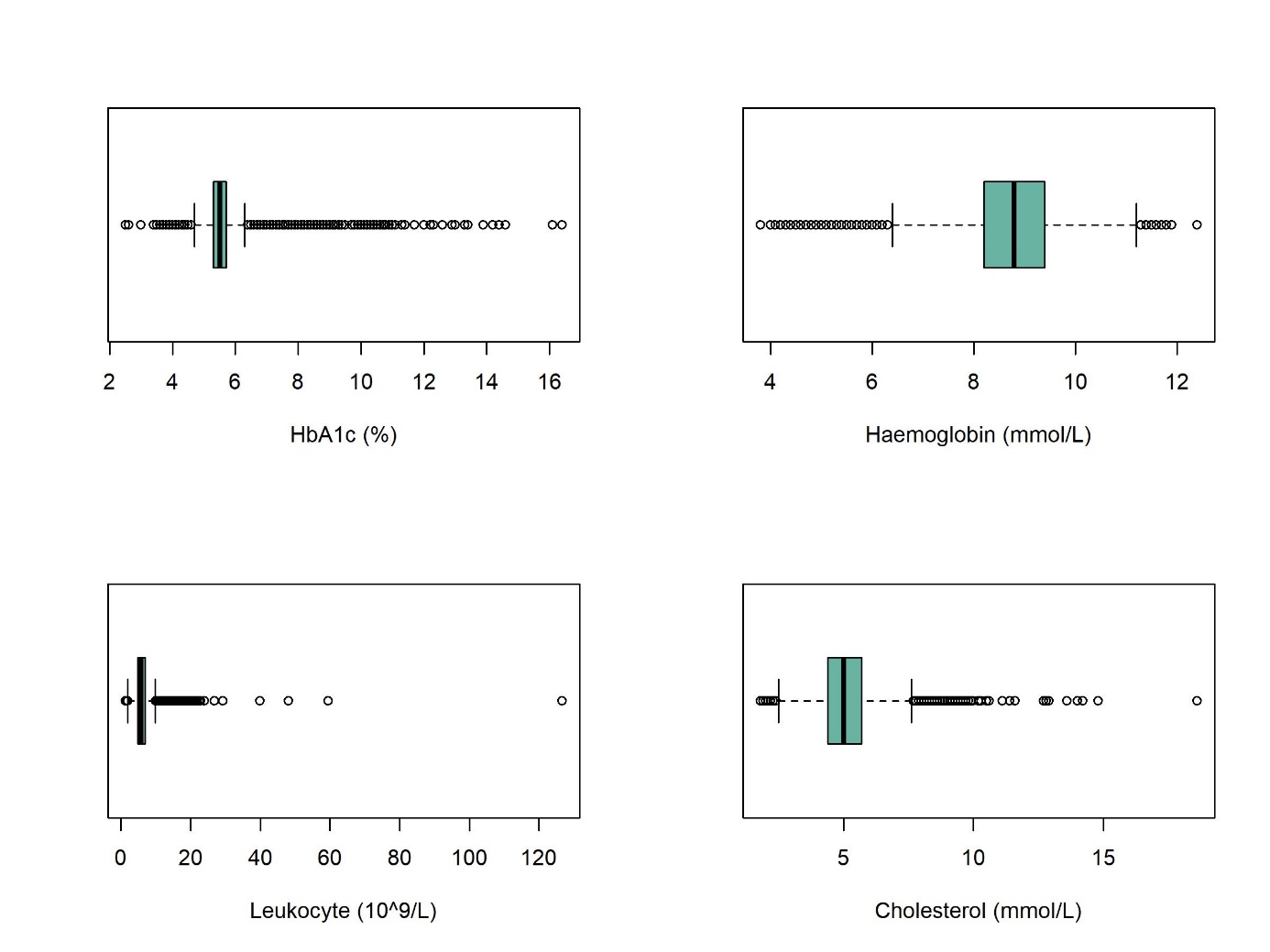

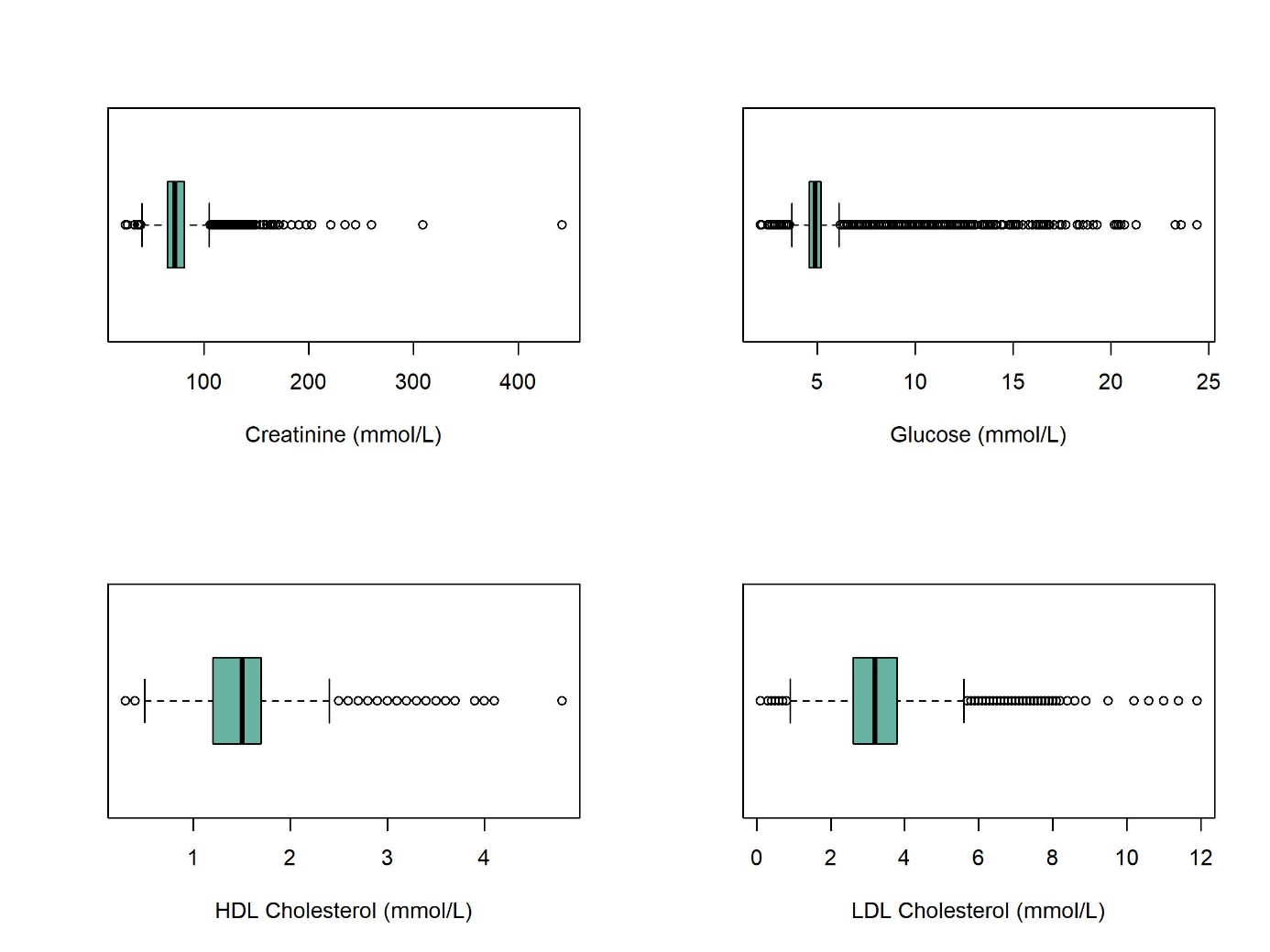

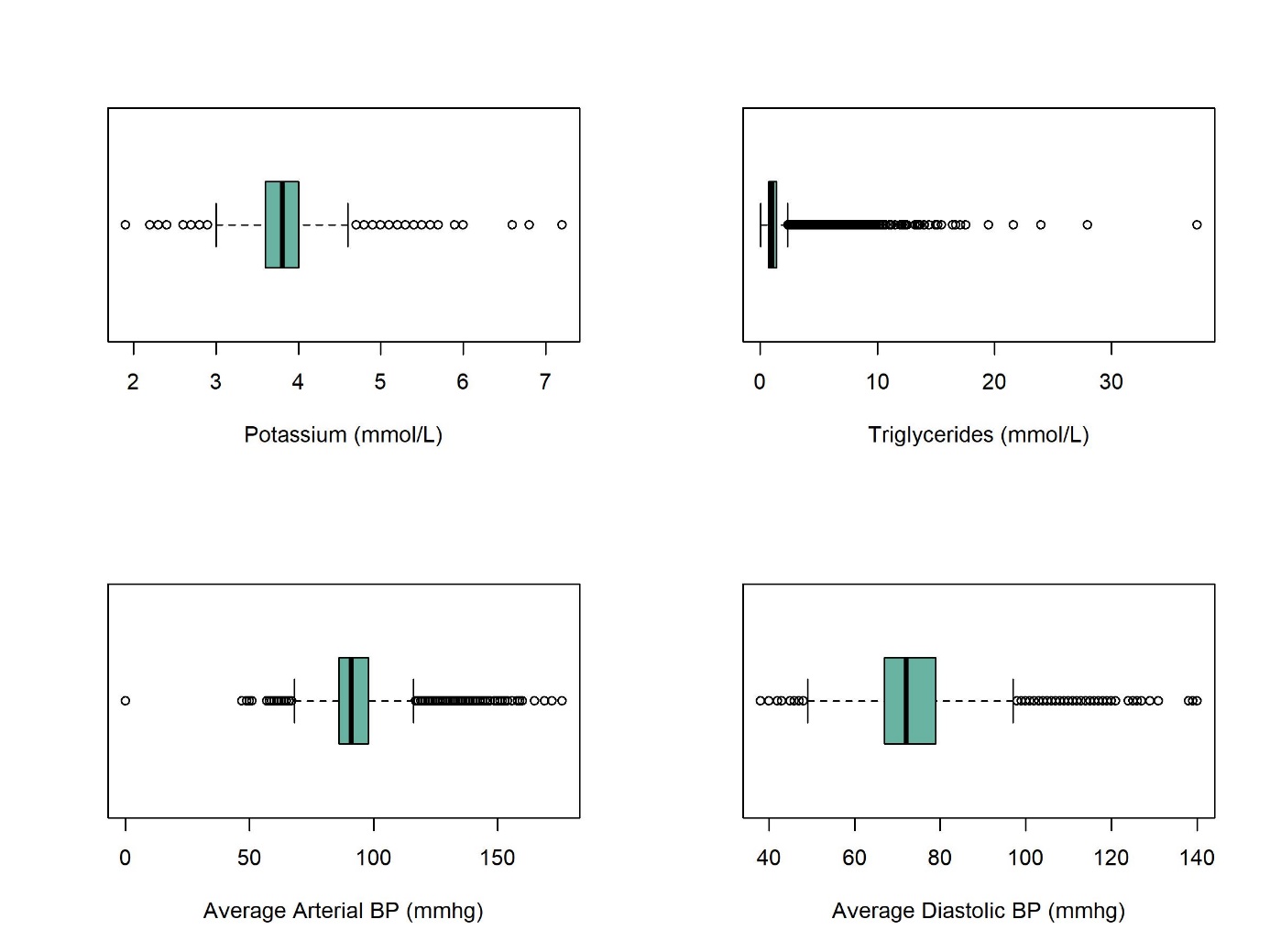

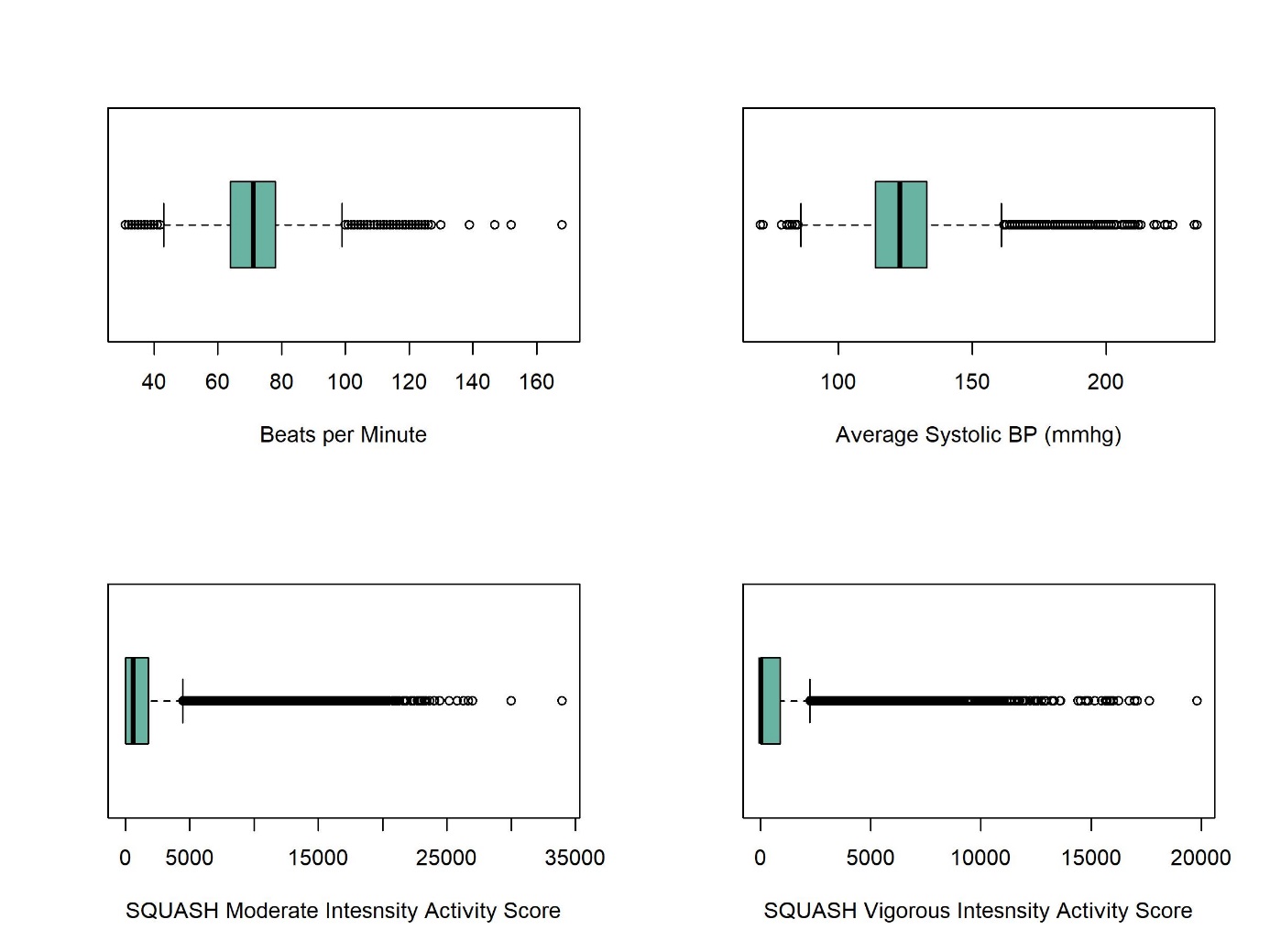


Figure S1: Boxplots used to identify outliers and those with chronic disease states that were not identified by the questionnaire.

**Poisson plots**
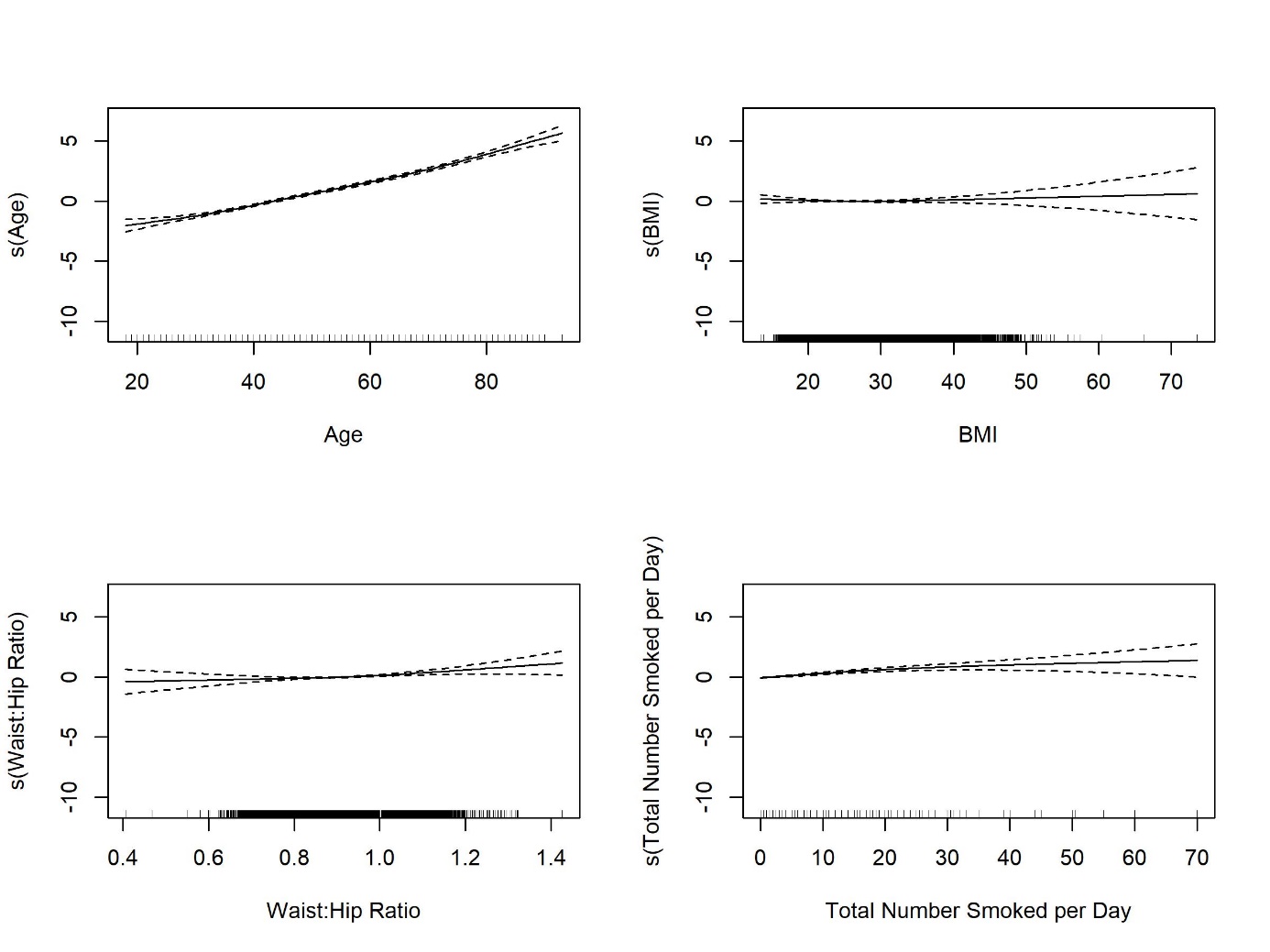

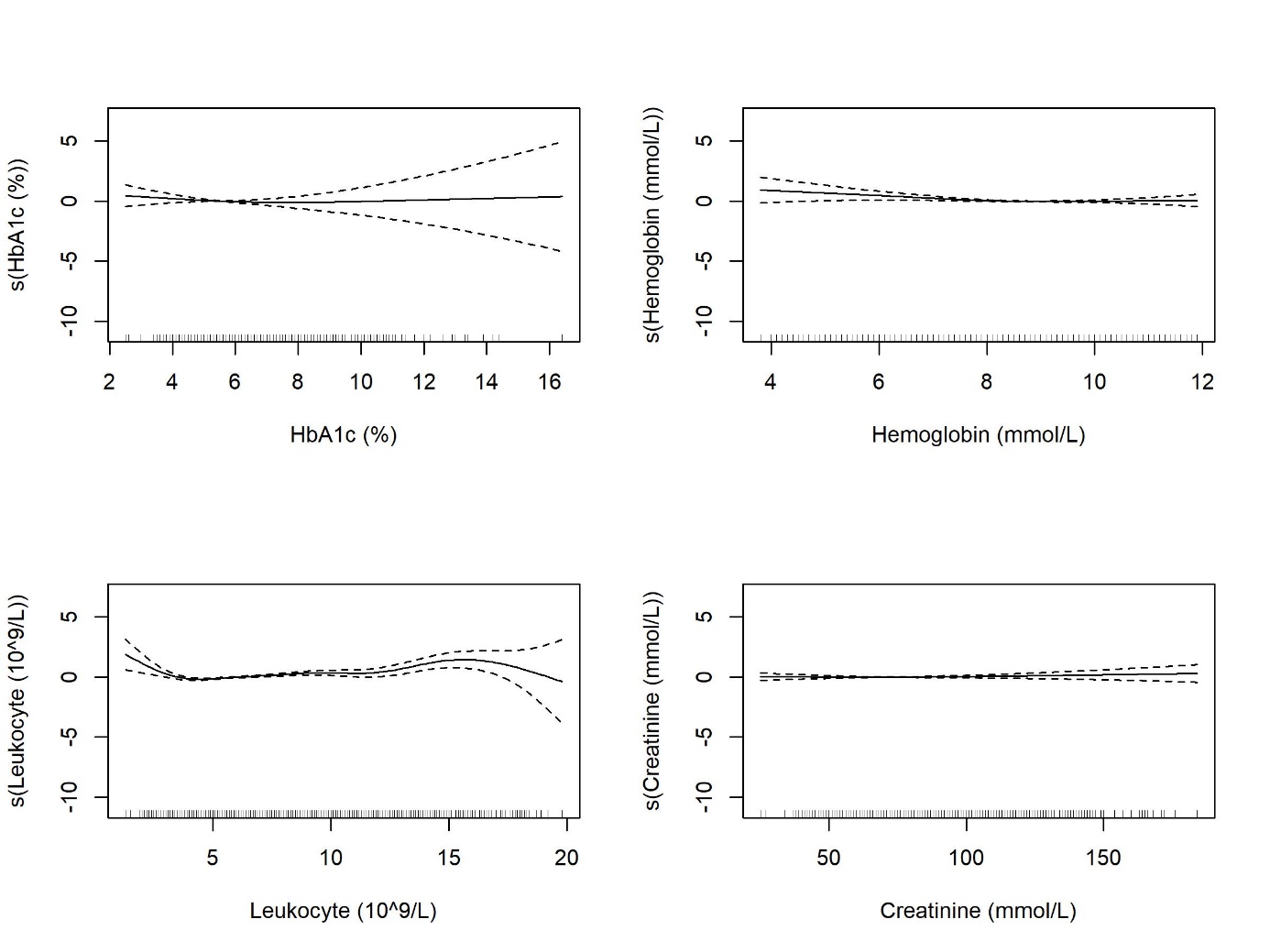

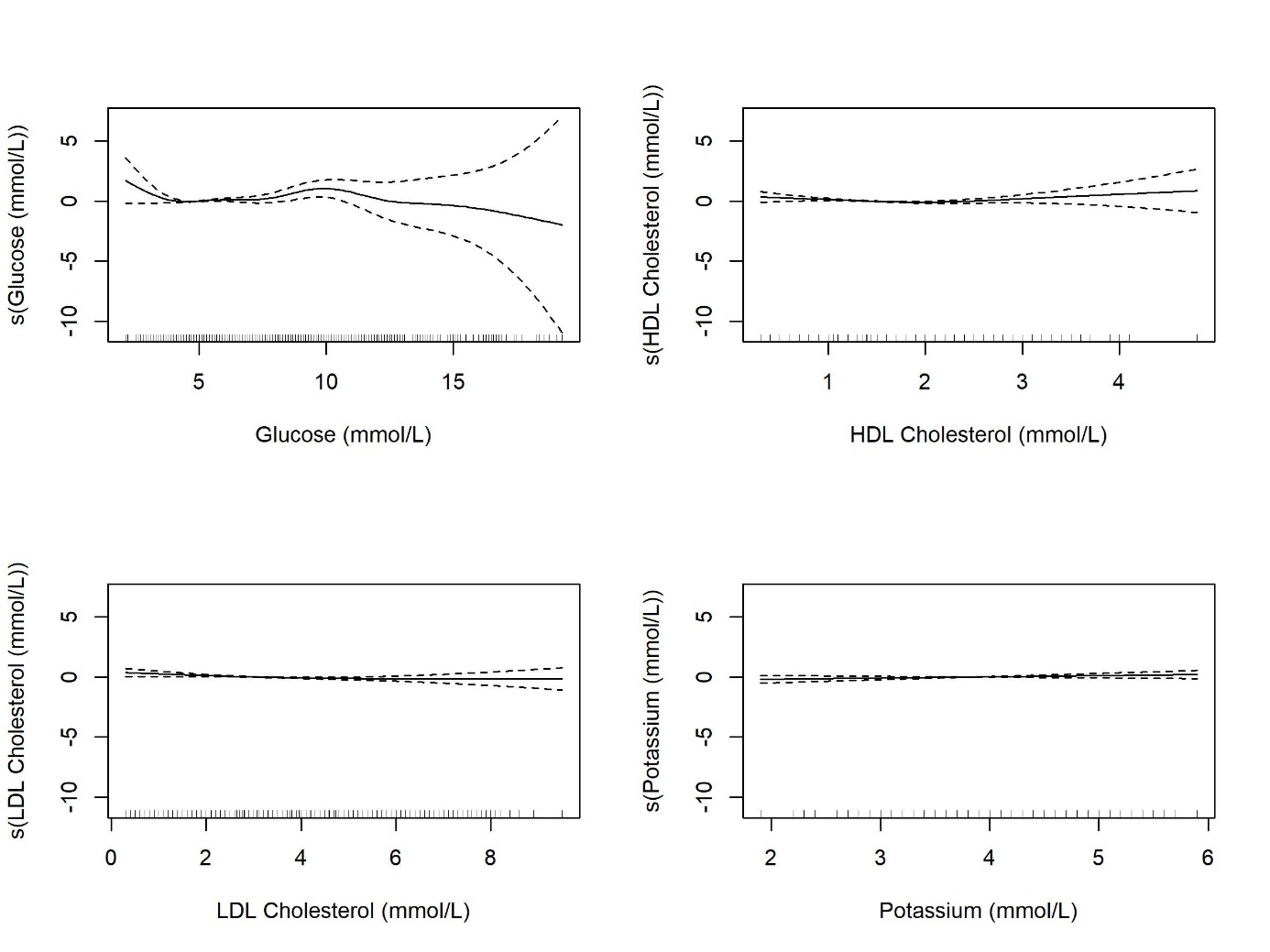

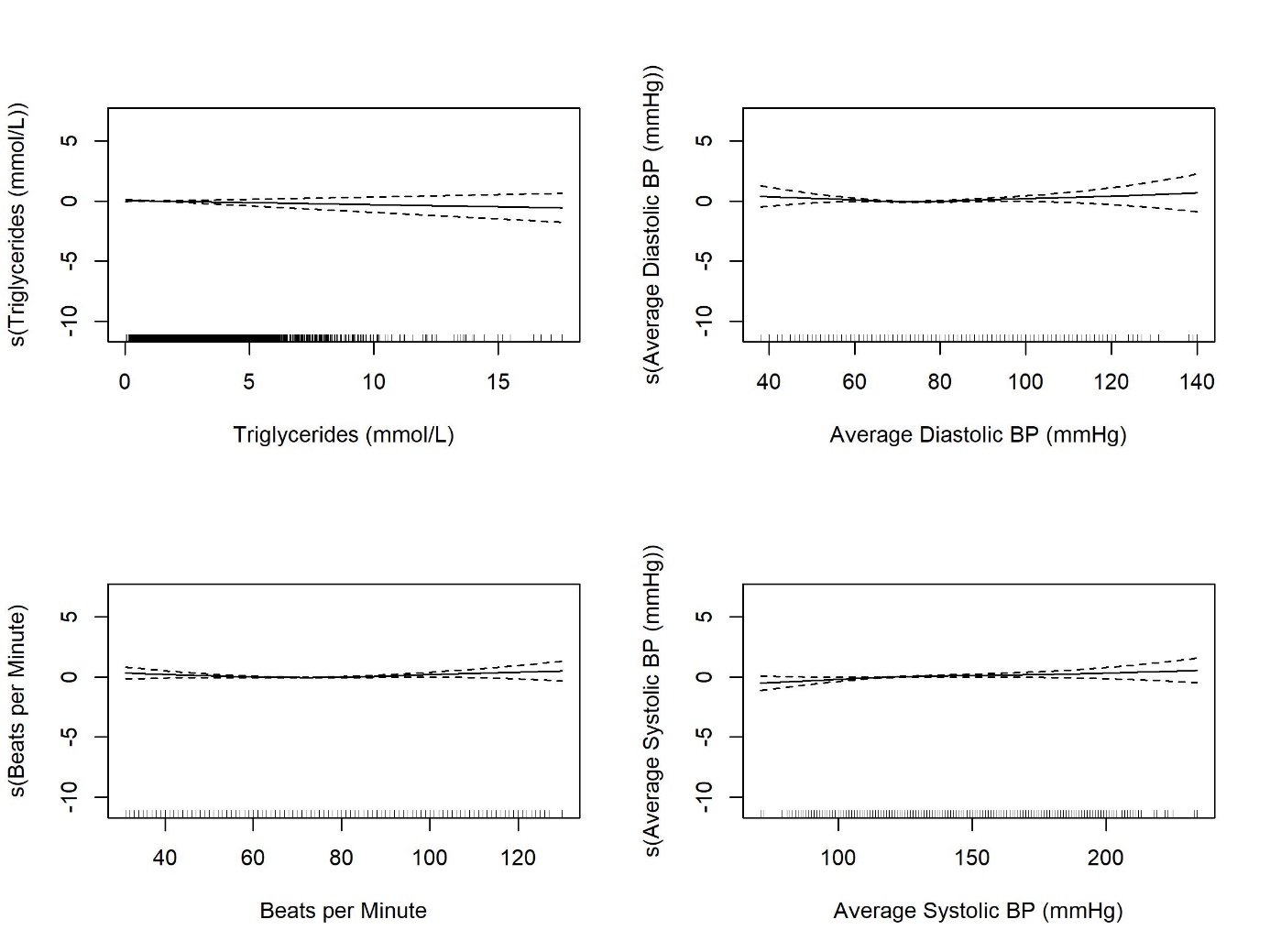

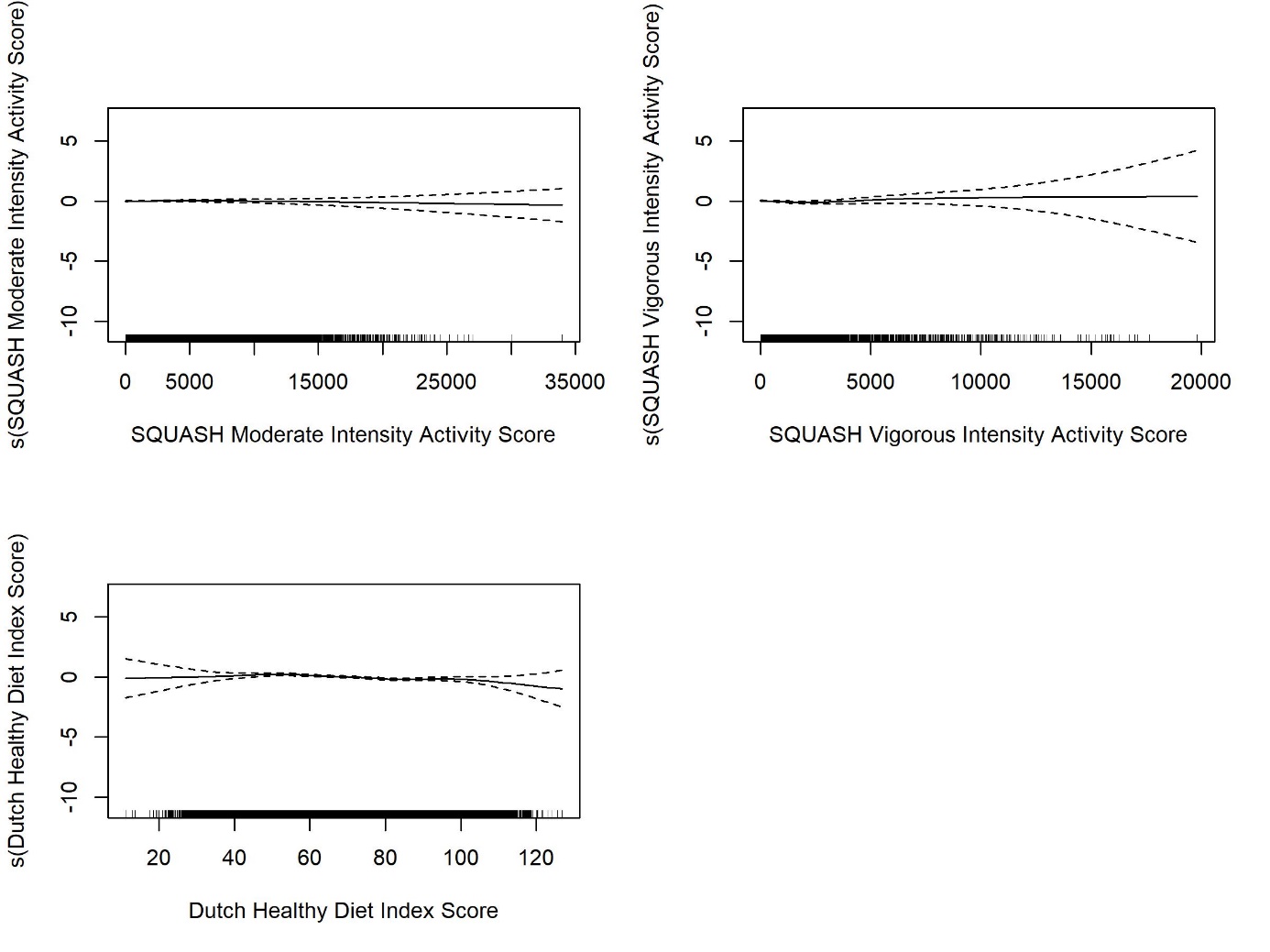
Figure S2: Poisson plots of each of the continuous variables are shown and used to estimate the relationship between predictors and the outcome variable.

**Penalized splines for testing non-linearity**

Table S5: The Cox regression summary output after the addition of splines to all continuous variables to test for nonlinearity. Only continuous variables are shown, although all variables were included in the model.

| **Variable (Linear & Nonlinear terms)** | **Coefficient** | **se(Coefficient)** | **Chi-squared** | **df** | **p** |
| --- | --- | --- | --- | --- | --- |
| *pspline Age Linear* | 0.0983 | 0.0029 | 1,122.0333 | 1.0000 | 0.0000 |
| *pspline Age Nonlinear* |  |  | 10.9616 | 3.0986 | 0.0130 |
| *pspline BMI Linear* | 0.0035 | 0.0075 | 0.2102 | 1.0000 | 0.6466 |
| *pspline BMI Nonlinear* |  |  | 2.5718 | 3.0010 | 0.4626 |
| *pspline Waist: Hip Ratio Linear* | 0.7228 | 0.4312 | 2.8098 | 1.0000 | 0.0937 |
| *pspline Waist: Hip Ratio Nonlinear* |  |  | 5.7332 | 3.0017 | 0.1255 |
| *pspline Total Number Smoked per Day Linear* | 0.0274 | 0.0067 | 16.7753 | 1.0000 | 0.0000 |
| *pspline Total Number Smoked per Day Nonlinear* |  |  | 4.7862 | 3.0046 | 0.1886 |
| *pspline HbA1c (%) Linear* | -0.0823 | 0.0807 | 1.0403 | 1.0000 | 0.3077 |
| *pspline HbA1c (%) Nonlinear* |  |  | 2.4553 | 3.0001 | 0.4834 |
| *pspline Haemoglobin (mmol/L) Linear* | -0.1092 | 0.0416 | 6.8976 | 1.0000 | 0.0086 |
| *pspline Haemoglobin (mmol/L) Nonlinear* |  |  | 5.2992 | 3.0098 | 0.1521 |
| *pspline Leukocyte (10^9/L) Linear* | 0.0822 | 0.0153 | 28.9416 | 1.0000 | 0.0000 |
| *pspline Leukocyte (10^9/L) Nonlinear* |  |  | 5.8486 | 3.0420 | 0.1226 |
| *pspline Creatinine (mmol/L) Linear* | -0.0014 | 0.0021 | 0.4074 | 1.0000 | 0.5233 |
| *pspline Creatinine (mmol/L) Nonlinear* |  |  | 5.2728 | 3.0289 | 0.1557 |
| *pspline Glucose (mmol/L) Linear* | 0.0559 | 0.0448 | 1.5530 | 1.0000 | 0.2127 |
| *pspline Glucose (mmol/L) Nonlinear* |  |  | 4.2262 | 3.0001 | 0.2381 |
| *pspline HDL Cholesterol (mmol/L) Linear* | -0.04 | 0.0769 | 0.2702 | 1.0000 | 0.6032 |
| *pspline HDL Cholesterol (mmol/L) Nonlinear* |  |  | 7.5684 | 3.0000 | 0.0558 |
| *pspline LDL Cholesterol (mmol/L) Linear* | -0.065 | 0.0301 | 4.6672 | 1.0000 | 0.0307 |
| *pspline LDL Cholesterol (mmol/L) Nonlinear* |  |  | 3.6442 | 3.0220 | 0.3059 |
| *pspline Potassium (mmol/L) Linear* | 0.0989 | 0.0817 | 1.4662 | 1.0000 | 0.2259 |
| *pspline Potassium (mmol/L) Nonlinear* |  |  | 1.0867 | 3.0005 | 0.7804 |
| *pspline Triglycerides (mmol/L) Linear* | -0.02 | 0.0371 | 0.2921 | 1.0000 | 0.5889 |
| *pspline Triglycerides (mmol/L) Nonlinear* |  |  | 2.8197 | 3.0001 | 0.4203 |
| *pspline Average Diastolic BP (mmHg) Linear* | 0.0022 | 0.0036 | 0.3610 | 1.0000 | 0.5480 |
| *pspline Average Diastolic BP (mmHg) Nonlinear* |  |  | 10.7300 | 2.9986 | 0.0133 |
| *pspline Beats per Minute Linear* | 5e-04 | 0.0023 | 0.0556 | 1.0000 | 0.8137 |
| *pspline Beats per Minute Nonlinear* |  |  | 8.6620 | 3.0250 | 0.0348 |
| *pspline Average Systolic BP (mmHg) Linear* | 0.0053 | 0.0022 | 5.9694 | 1.0000 | 0.0146 |
| *pspline Average Systolic BP (mmHg) Nonlinear* |  |  | 4.3121 | 3.0286 | 0.2333 |
| *pspline SQUASH Moderate Intensity Activity Score Linear* | 0 | 0 | 0.0000 | 1.0000 | 0.9949 |
| *pspline SQUASH Moderate Intensity Activity Score Nonlinear* |  |  | 4.5674 | 3.0069 | 0.2072 |
| *pspline SQUASH Vigorous Intensity Activity Score Linear* | 0 | 0 | 0.3904 | 1.0000 | 0.5321 |
| *pspline SQUASH Vigorous Intensity Activity Score Nonlinear* |  |  | 4.6289 | 2.9998 | 0.2011 |
| *pspline Dutch Healthy Diet Index Score Linear* | -0.0086 | 0.0019 | 19.7551 | 1.0000 | 0.0000 |
| *pspline Dutch Healthy Diet Index Score Nonlinear* |  |  | 4.4224 | 3.0251 | 0.2224 |

Table S6: The proportional hazards test outcome of the final model model

| **Variable** | **Chi-squared** | **df** | **p** |
| --- | --- | --- | --- |
| *pspline Age* | 10.9530 | 4.0879753 | 0.0289 |
| *pspline BMI* | 1.6869 | 2.0001461 | 0.4303 |
| *pspline Waist: Hip Ratio* | 0.1813 | 2.0001784 | 0.9134 |
| *pspline Total Number Smoked per Day* | 0.4914 | 2.0010877 | 0.7824 |
| *pspline HbA1c (%)* | 2.3904 | 1.9999809 | 0.3026 |
| *pspline Haemoglobin (mmol/L)* | 1.5244 | 2.0017083 | 0.4671 |
| *pspline Leukocyte (10^9/L)* | 1.3796 | 4.0337990 | 0.8512 |
| *pspline HDL Cholesterol (mmol/L)* | 6.6406 | 4.0000469 | 0.1562 |
| *pspline LDL Cholesterol (mmol/L)* | 6.9184 | 4.0182123 | 0.1417 |
| *pspline Triglycerides (mmol/L)* | 0.0002 | 1.9999982 | 0.9999 |
| *pspline Average Diastolic BP (mmHg)* | 0.4134 | 2.0008256 | 0.8134 |
| *pspline Beats per Minute* | 1.8220 | 2.0040585 | 0.4030 |
| *pspline Average Systolic BP (mmHg)* | 0.7280 | 2.0021632 | 0.6954 |
| *pspline SQUASH Moderate Intensity Activity Score* | 0.0372 | 2.0010777 | 0.9816 |
| *pspline SQUASH Vigorous Intensity Activity Score* | 0.3022 | 2.0002153 | 0.8598 |
| *pspline Dutch Healthy Diet Index Score* | 3.4904 | 2.0068417 | 0.1755 |
| *Gender (Female)* | 2.5201 | 0.9861020 | 0.1103 |
| *Current Smoker* | 0.0185 | 0.8352762 | 0.8409 |
| *Ex-Smoker* | 0.2041 | 0.9983838 | 0.6507 |
| *Creatinine (mmol/L)* | 0.2177 | 0.9970745 | 0.6396 |
| *Glucose (mmol/L)* | 0.0290 | 0.9710131 | 0.8560 |
| *Potassium (mmol/L)* | 0.0219 | 0.9971002 | 0.8816 |
| *Anemia (Yes)* | 0.4613 | 0.9980463 | 0.4962 |
| *COPD (No)* | 1.2111 | 0.9989833 | 0.2708 |
| *Highest Level of Education* | 6.9512 | 7.9885170 | 0.5407 |
| *Eating Disorder (Yes)* | 0.6555 | 0.9970382 | 0.4170 |
| *Gall Stones (Yes)* | 2.4740 | 0.9980766 | 0.1155 |
| *Hypertension (No)* | 3.1664 | 0.9967597 | 0.0748 |
| *I Live Alone (No)* | 0.5351 | 0.9969078 | 0.4633 |
| *How much of the time been nervous during the past 4wks?* | 1.2678 | 4.9960836 | 0.9380 |
| *How much of the time been calm & peaceful during the past 4wks?* | 2.5544 | 4.9966613 | 0.7679 |
| *How much of the time been downhearted & blue past 4wks?* | 2.1096 | 4.9961742 | 0.8334 |
| *How much of the time had a lot of energy during the past 4wks?* | 3.6499 | 4.9953007 | 0.6002 |
| *How much of the time feeling worn out during the past 4wks?* | 7.3895 | 4.9960530 | 0.1929 |
| *How much of the time feeling tired during the past 4wks?* | 5.2284 | 4.9959926 | 0.3881 |
| *How would you rate your health, generally speaking?* | 3.3295 | 3.9950351 | 0.5035 |
| *Physical health limited work during past 4wks?* | 1.4988 | 0.9992433 | 0.2207 |
| *I expect my health to get worse* | 3.0272 | 3.9949865 | 0.5525 |
| *My health is excellent* | 2.4912 | 3.9964768 | 0.6457 |
| *Sleep* | 6.2163 | 5.9894439 | 0.3982 |
| ***Full Model*** | **76.3080** | **108.8690449** | **0.9924** |


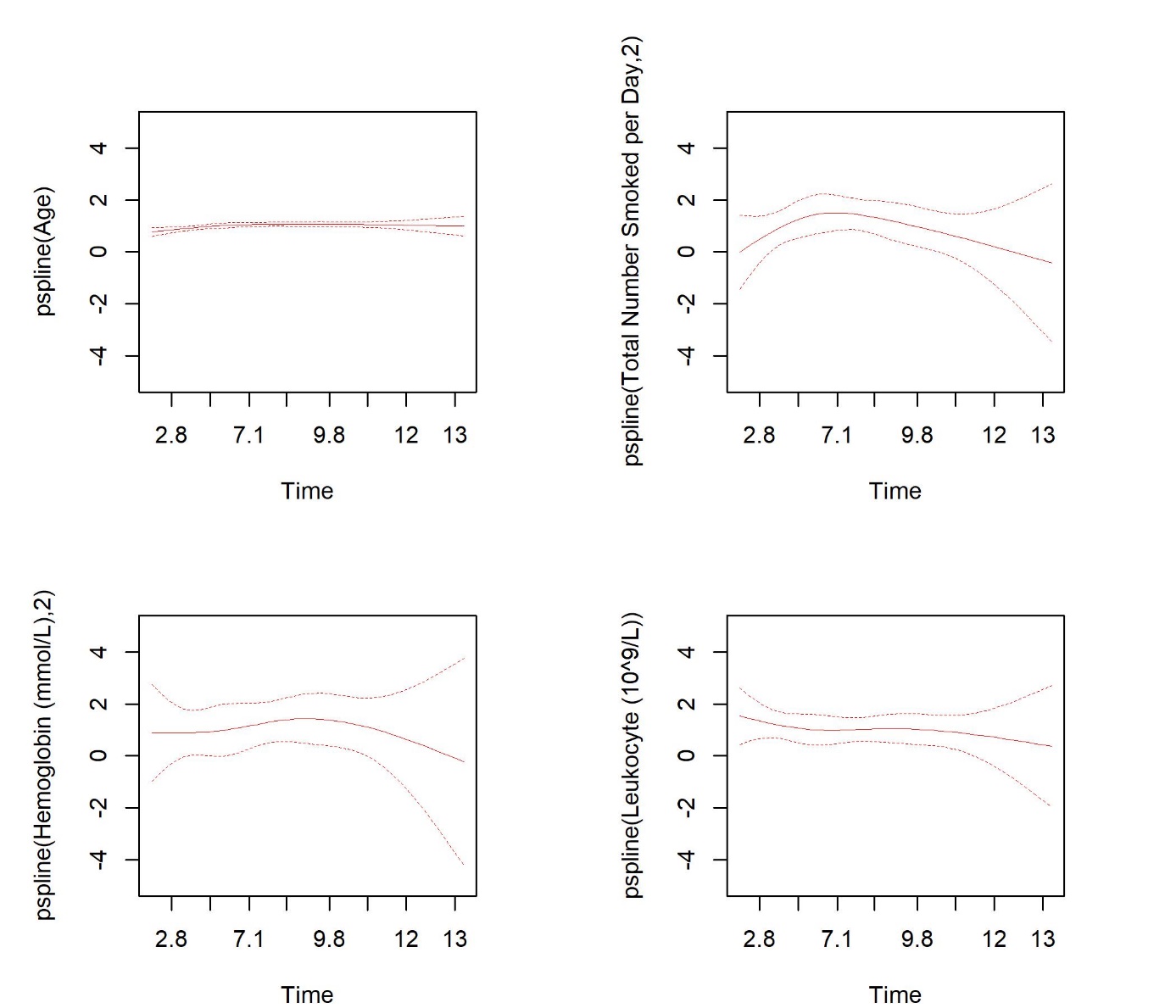

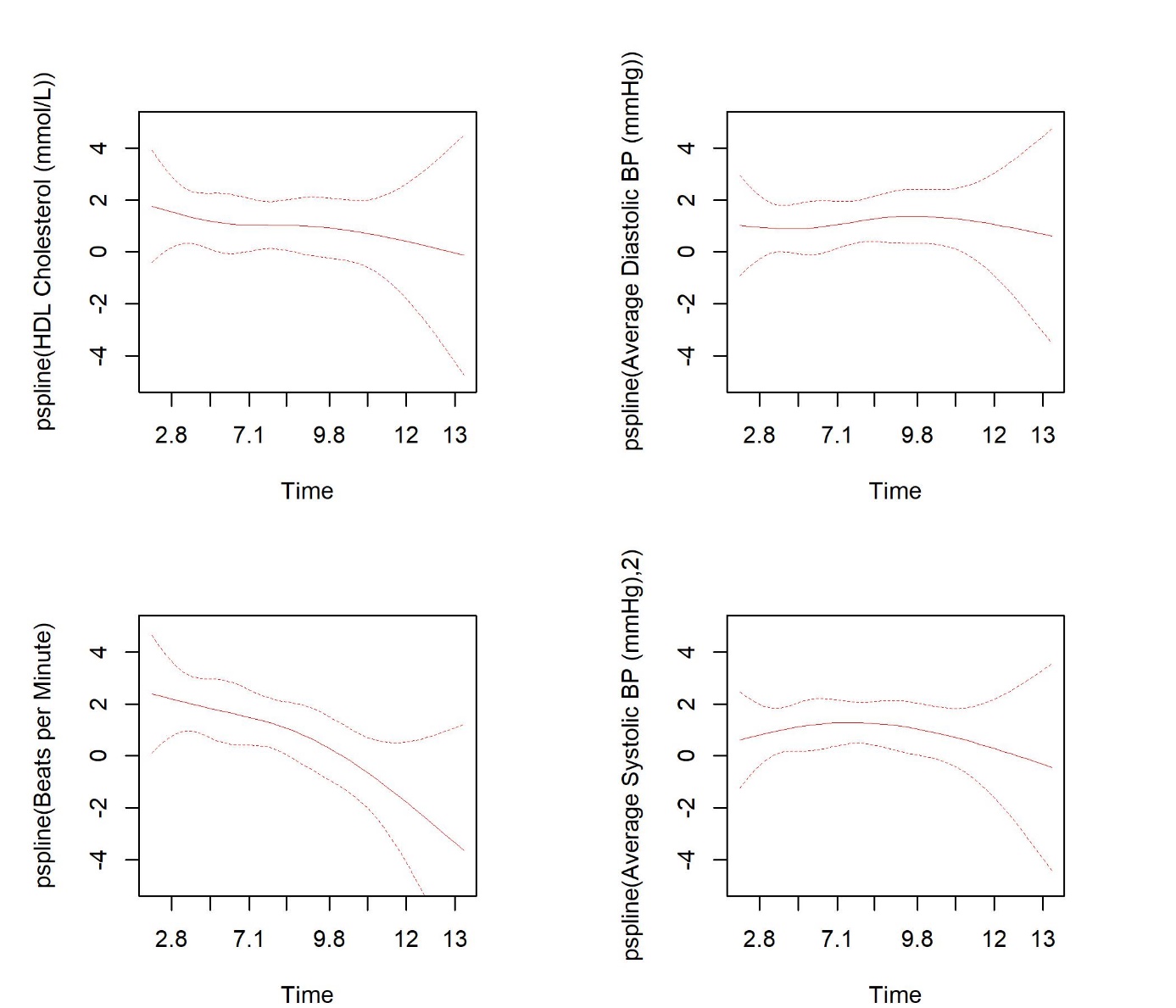

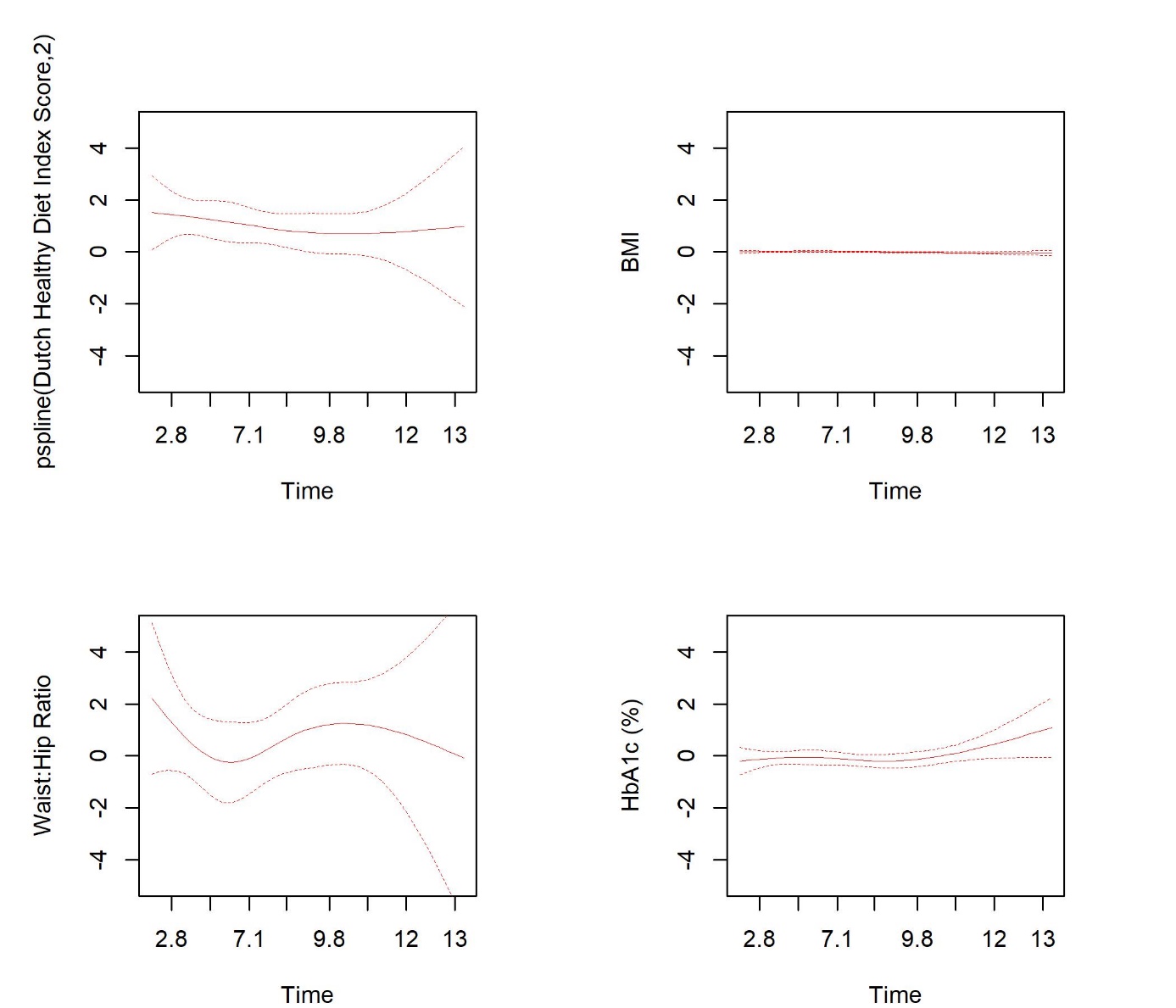

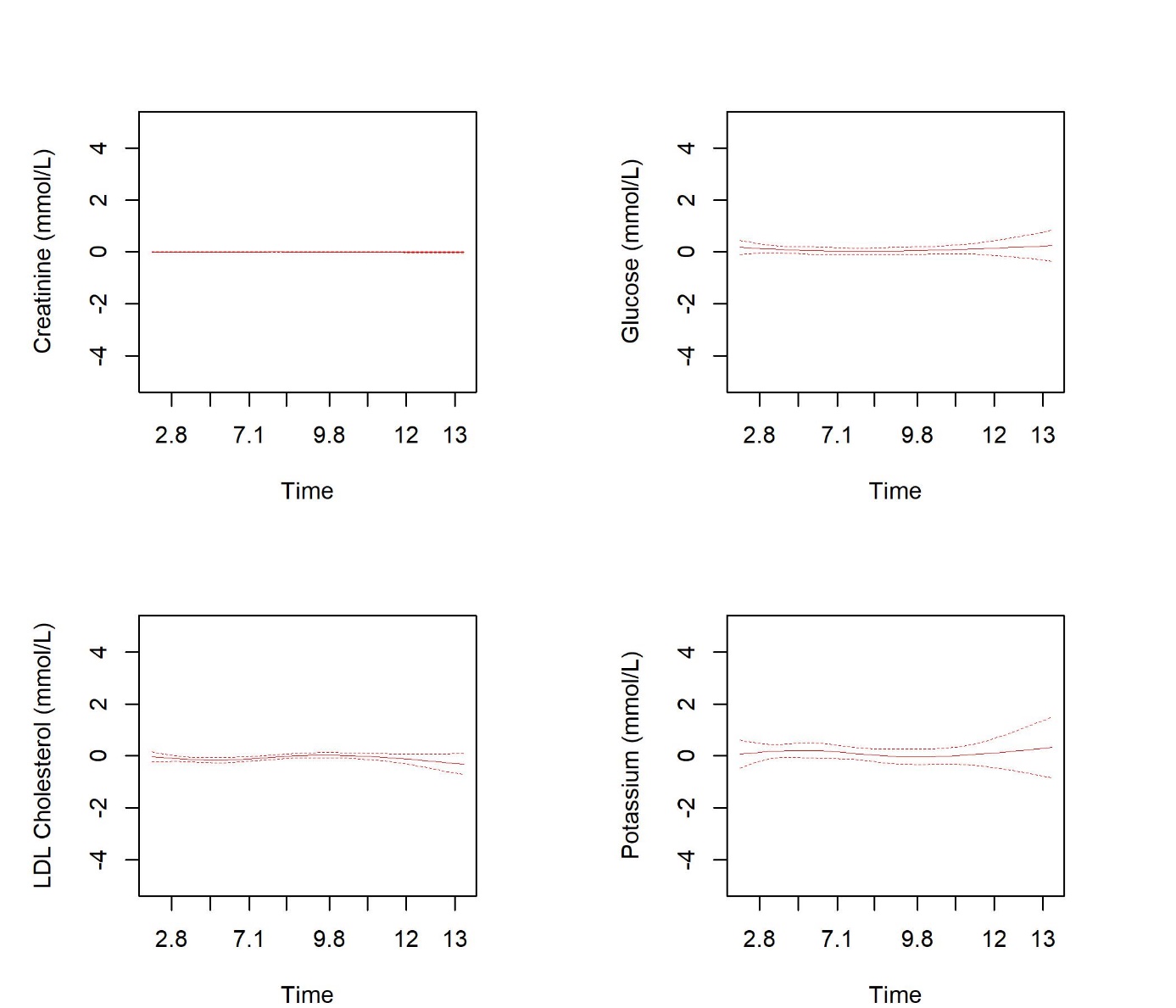

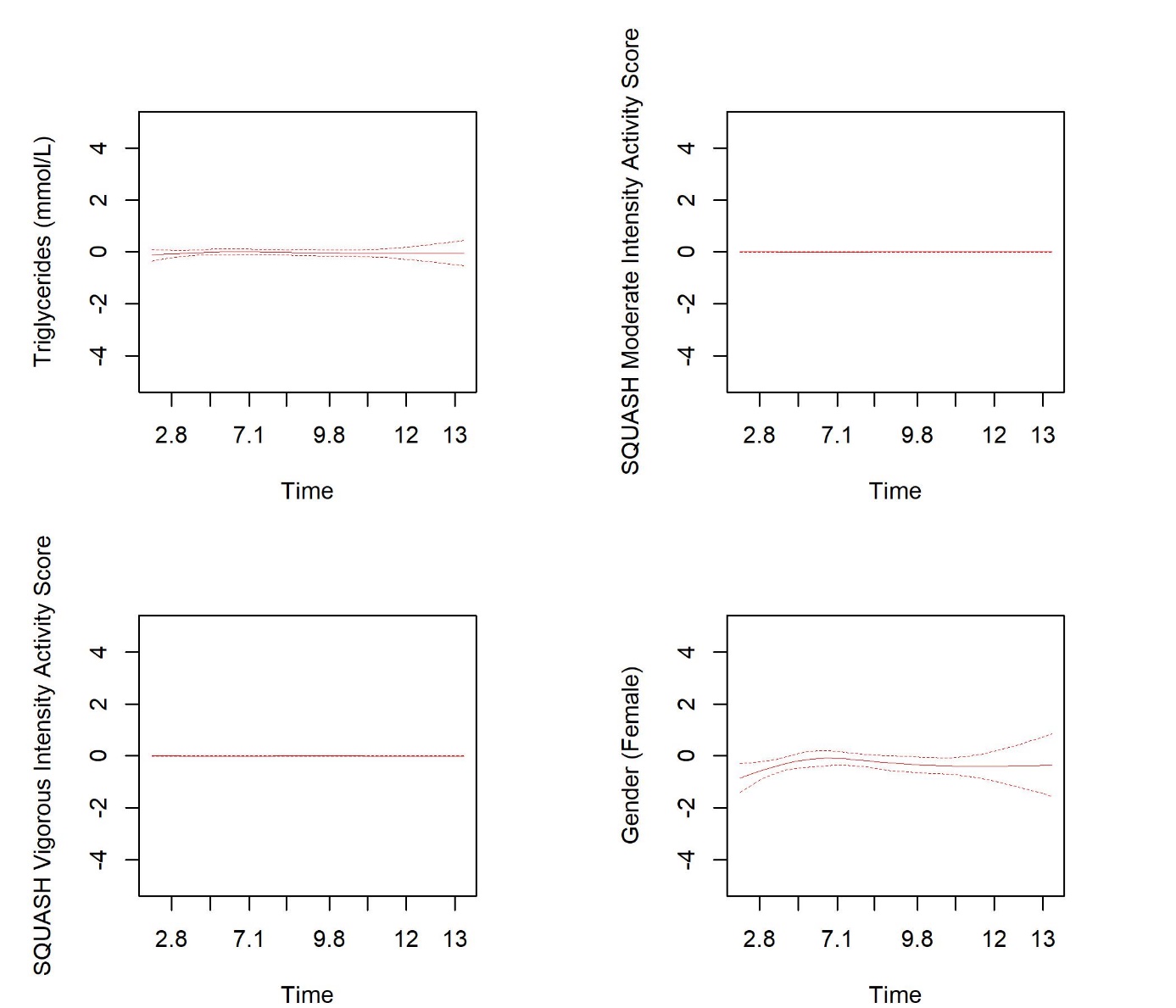

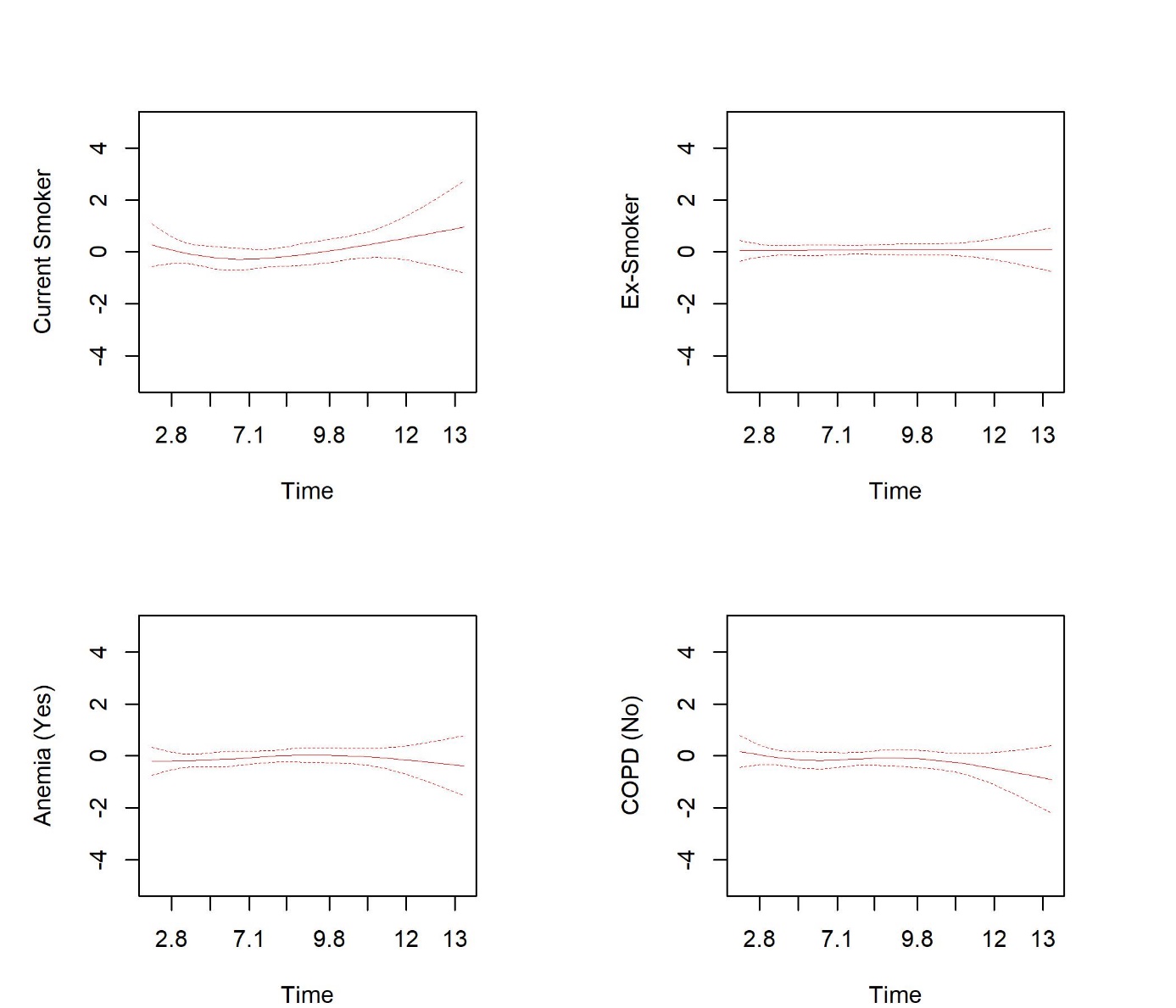

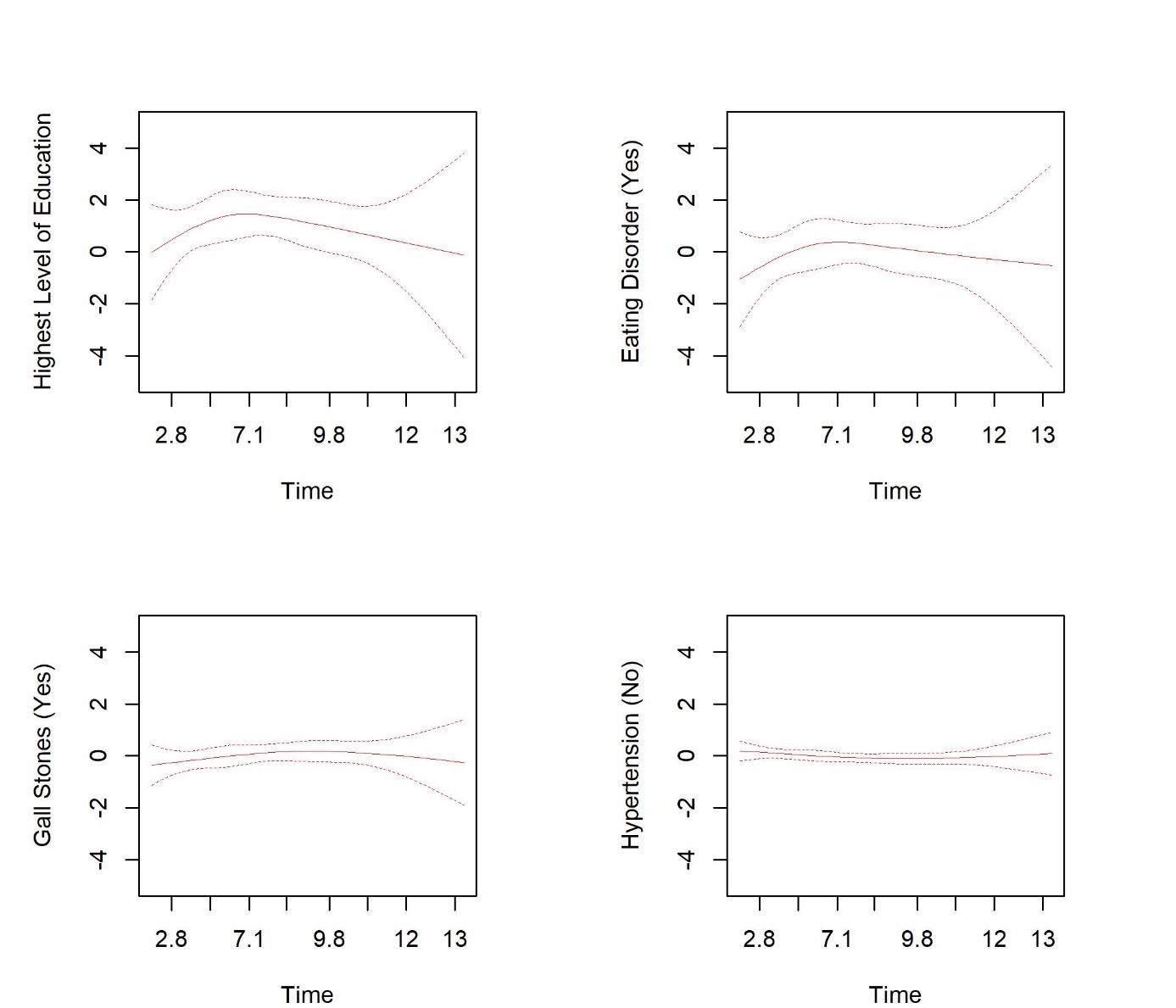

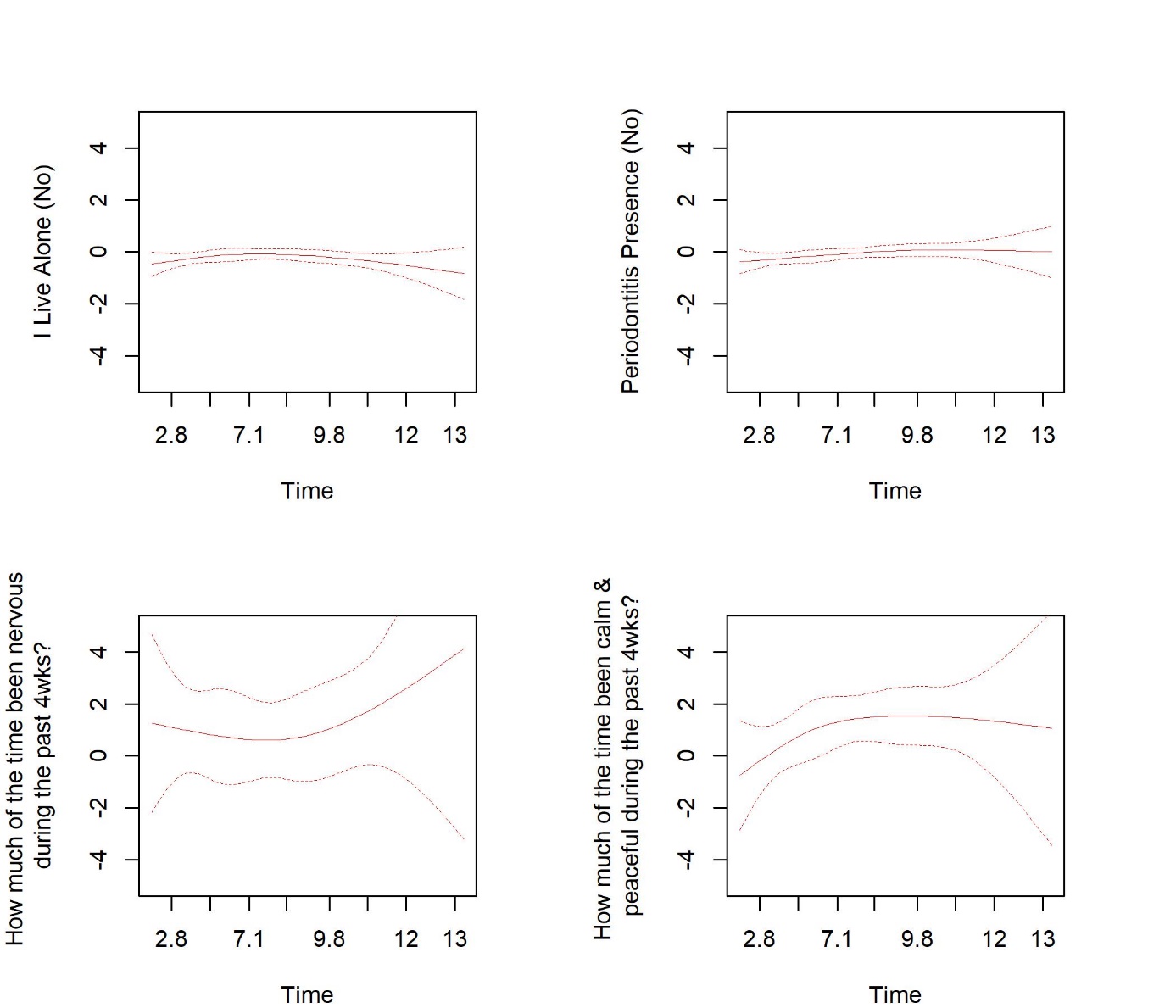

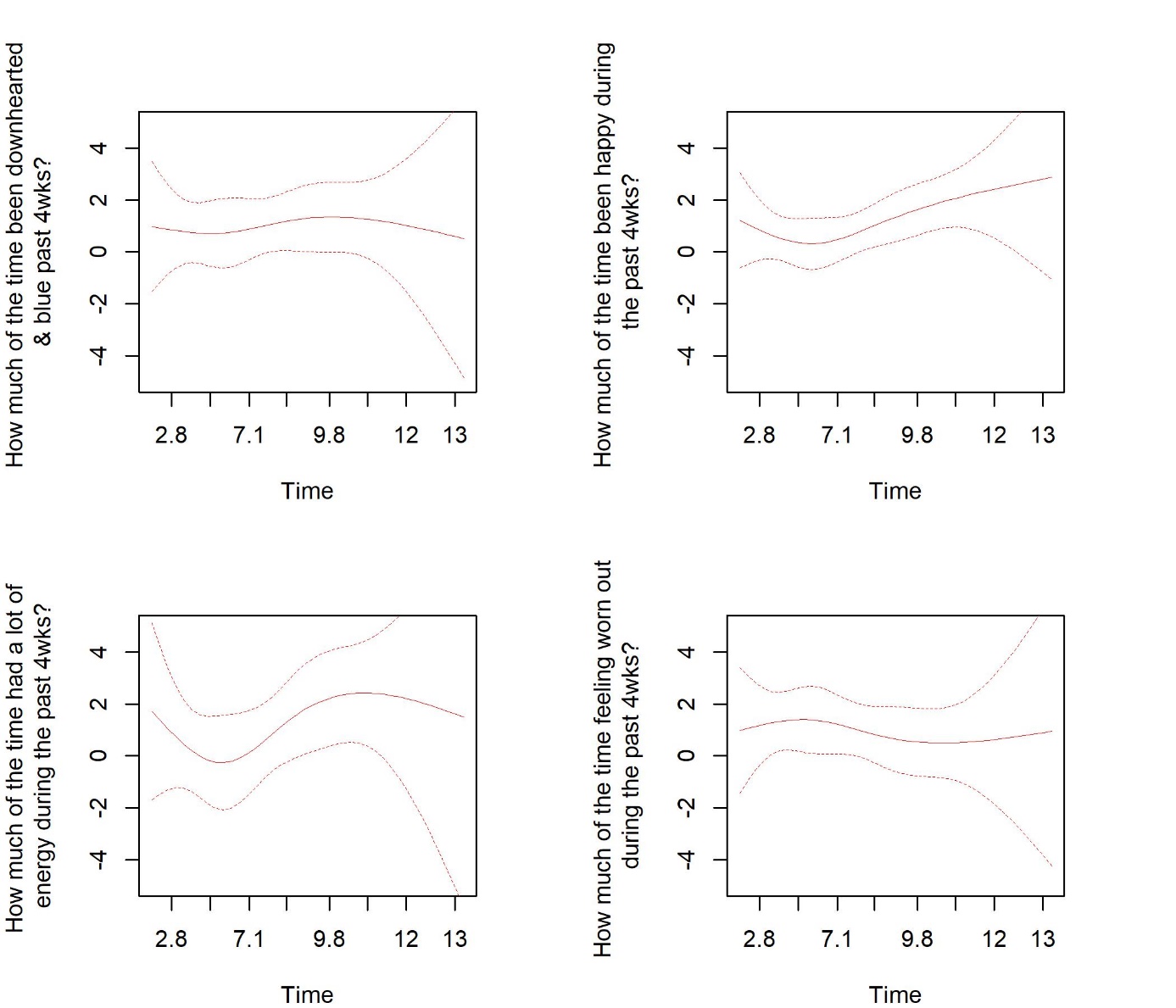

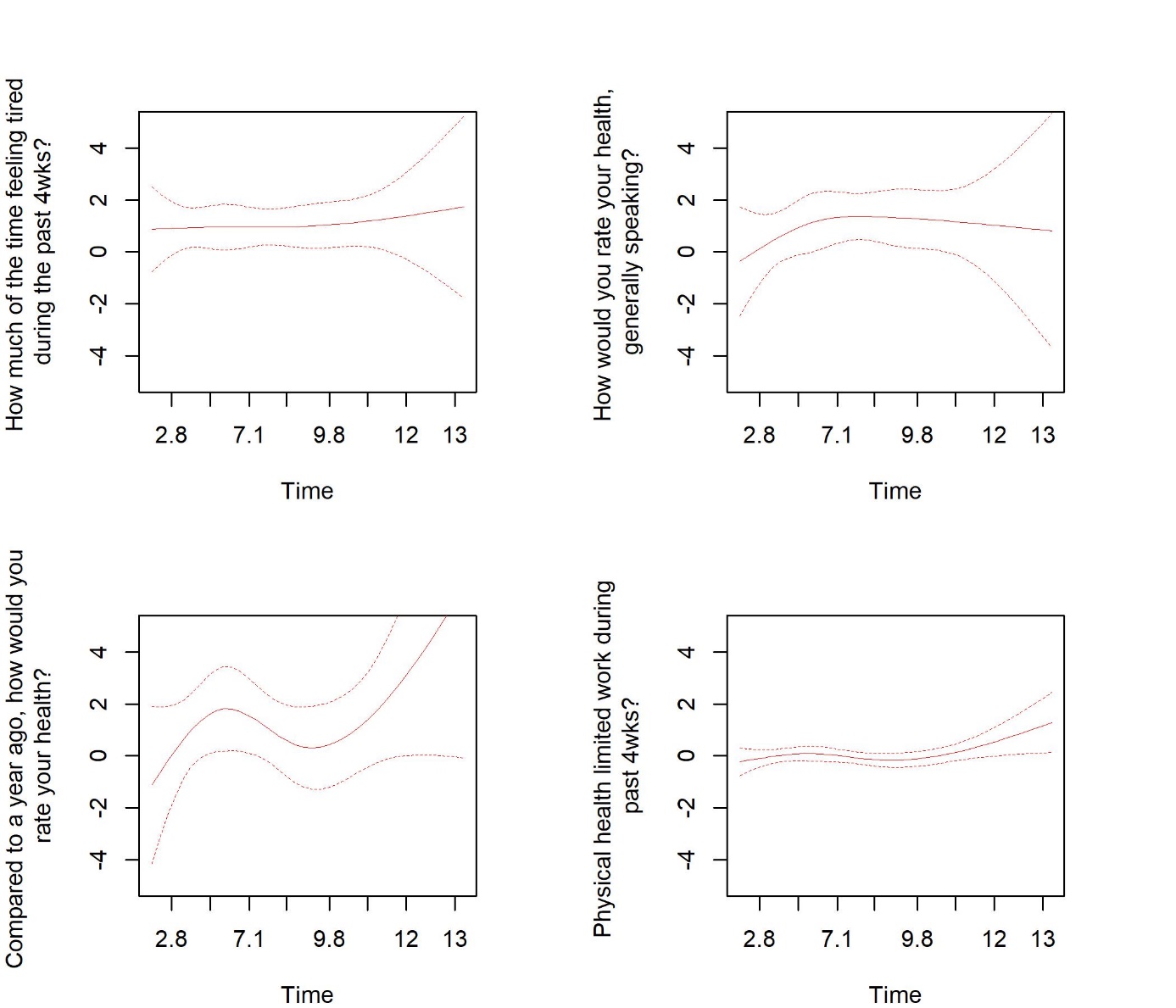

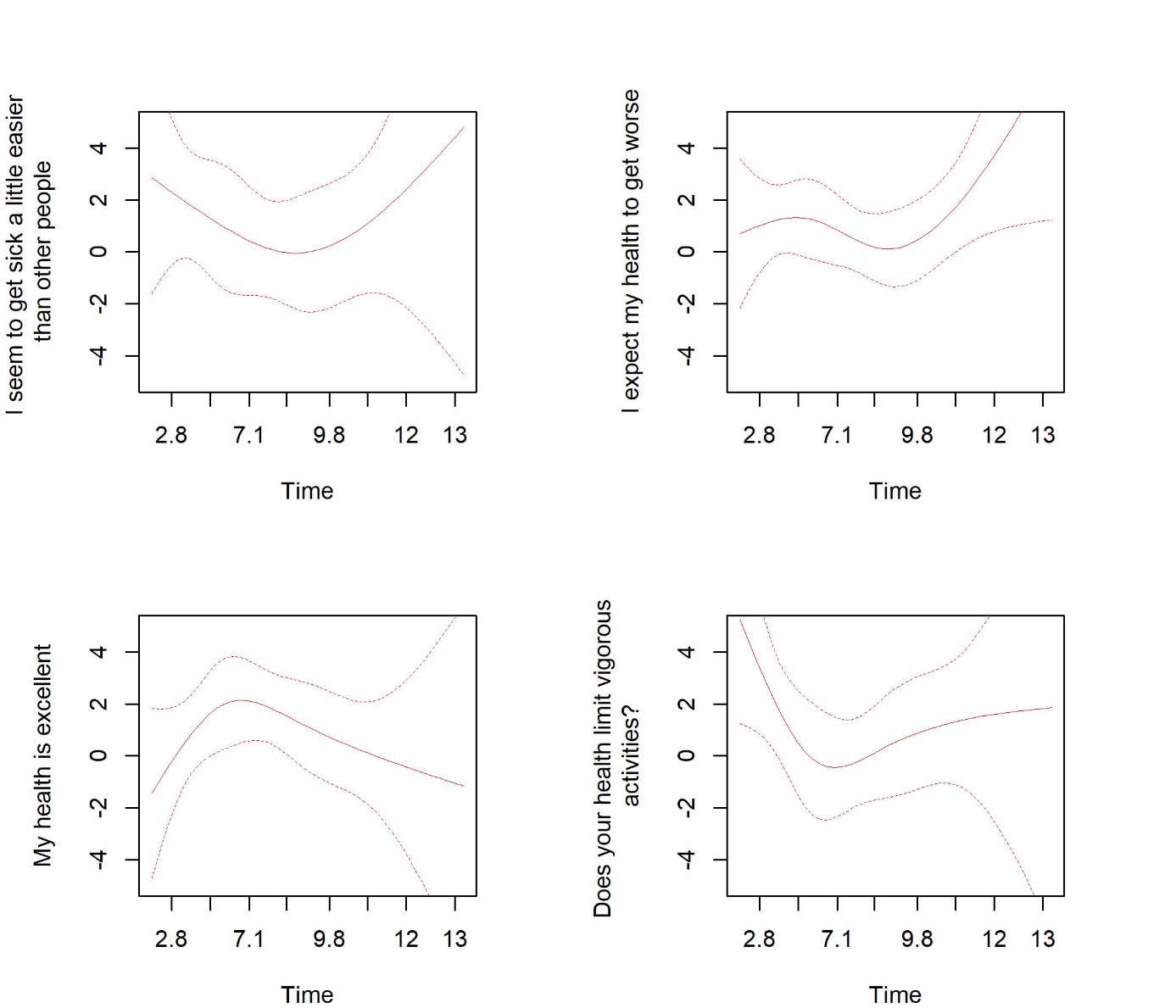

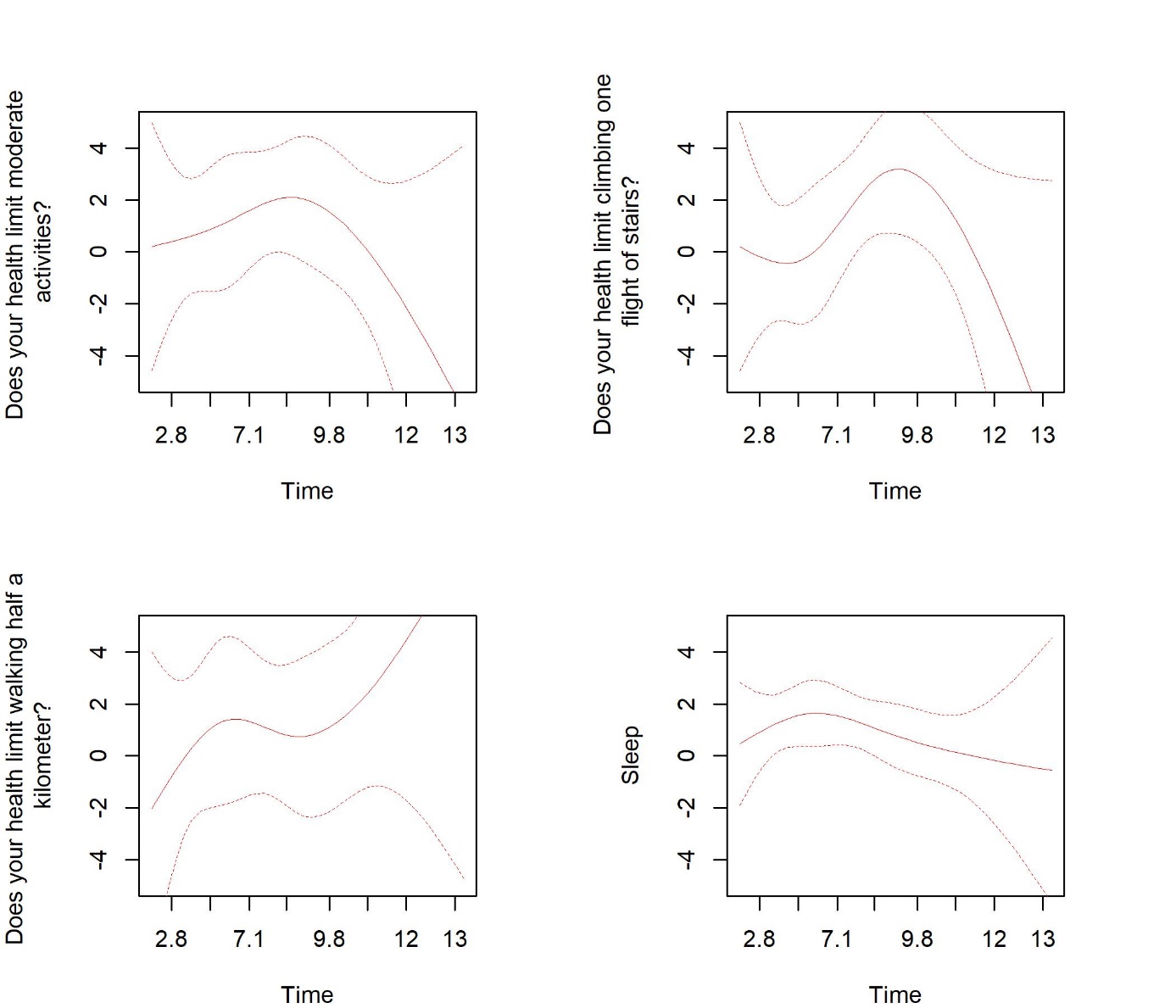
Figure S3: Schoenfeld plots of all variables included in the final model.

**Regression summary of the final model**

Table S7: The Cox summary output and relevant test statistics of the final model.

| **Variable** | **Coefficient** | **se(Coefficient)** | **Chi-squared** | **df** | **p** |
| --- | --- | --- | --- | --- | --- |
| *pspline Age Linear* | 0.0986 | 0.0031 | 986.1192 | 1.0000 | 0.0000 |
| *pspline Age Nonlinear* |  |  | 6.8068 | 3.0880 | 0.0834 |
| *pspline BMI Linear* | 0.0035 | 0.0083 | 0.1770 | 1.0000 | 0.6740 |
| *pspline BMI Nonlinear* |  |  | 0.4994 | 1.0001 | 0.4798 |
| *pspline Waist: Hip Ratio Linear* | 0.5402 | 0.4642 | 1.3543 | 1.0000 | 0.2445 |
| *pspline Waist: Hip Ratio Nonlinear* |  |  | 2.8330 | 1.0002 | 0.0924 |
| *pspline Total Number Smoked per Day Linear* | 0.0280 | 0.0070 | 15.7521 | 1.0000 | 0.0001 |
| *pspline Total Number Smoked per Day Nonlinear* |  |  | 3.1474 | 1.0011 | 0.0762 |
| *pspline HbA1c (%) Linear* | -0.1419 | 0.0871 | 2.6532 | 1.0000 | 0.1033 |
| *pspline HbA1c (%) Nonlinear* |  |  | 0.4694 | 1.0000 | 0.4932 |
| *pspline Haemoglobin (mmol/L) Linear* | -0.1275 | 0.0451 | 7.9892 | 1.0000 | 0.0047 |
| *pspline Haemoglobin (mmol/L) Nonlinear* |  |  | 2.5013 | 1.0017 | 0.1140 |
| *pspline Leukocyte (10^9/L) Linear* | 0.0880 | 0.0164 | 28.8193 | 1.0000 | 0.0000 |
| *pspline Leukocyte (10^9/L) Nonlinear* |  |  | 6.0723 | 3.0338 | 0.1106 |
| *pspline HDL Cholesterol (mmol/L) Linear* | -0.0557 | 0.0812 | 0.4694 | 1.0000 | 0.4933 |
| *pspline HDL Cholesterol (mmol/L) Nonlinear* |  |  | 12.3073 | 3.0000 | 0.0064 |
| *pspline LDL Cholesterol (mmol/L) Linear* | -0.0726 | 0.0314 | 5.3288 | 1.0000 | 0.0210 |
| *pspline LDL Cholesterol (mmol/L) Nonlinear* |  |  | 4.2698 | 3.0182 | 0.2361 |
| *pspline Triglycerides (mmol/L) Linear* | -0.0307 | 0.0423 | 0.5276 | 1.0000 | 0.4676 |
| *pspline Triglycerides (mmol/L) Nonlinear* |  |  | 0.8820 | 1.0000 | 0.3477 |
| *pspline Average Diastolic BP (mmHg) Linear* | 0.0054 | 0.0039 | 1.9780 | 1.0000 | 0.1596 |
| *pspline Average Diastolic BP (mmHg) Nonlinear* |  |  | 4.1401 | 1.0008 | 0.0419 |
| *pspline Beats per Minute Linear* | -0.0010 | 0.0025 | 0.1673 | 1.0000 | 0.6825 |
| *pspline Beats per Minute Nonlinear* |  |  | 6.5443 | 1.0041 | 0.0106 |
| *pspline Average Systolic BP (mmHg) Linear* | 0.0048 | 0.0023 | 4.2888 | 1.0000 | 0.0384 |
| *pspline Average Systolic BP (mmHg) Nonlinear* |  |  | 1.1386 | 1.0022 | 0.2866 |
| *pspline SQUASH Moderate Intensity Activity Score Linear* | 0.0000 | 0.0000 | 0.3147 | 1.0000 | 0.5748 |
| *pspline SQUASH Moderate Intensity Activity Score Nonlinear* |  |  | 2.3226 | 1.0011 | 0.1277 |
| *pspline SQUASH Vigorous Intensity Activity Score Linear* | 0.0000 | 0.0000 | 0.2563 | 1.0000 | 0.6127 |
| *pspline SQUASH Vigorous Intensity Activity Score Nonlinear* |  |  | 2.6388 | 1.0002 | 0.1043 |
| *pspline Dutch Healthy Diet Index Score Linear* | -0.0096 | 0.0021 | 21.3137 | 1.0000 | 0.0000 |
| *pspline Dutch Healthy Diet Index Score Nonlinear* |  |  | 1.6182 | 1.0068 | 0.2050 |
| *Gender (Female)* | -0.3056 | 0.0980 | 9.7158 | 1.0000 | 0.0018 |
| *Current Smoker* | 0.0092 | 0.1418 | 0.0042 | 1.0000 | 0.9483 |
| *Ex-Smoker* | 0.0647 | 0.0628 | 1.0601 | 1.0000 | 0.3032 |
| *Creatinine (mmol/L)* | -0.0029 | 0.0024 | 1.4865 | 1.0000 | 0.2228 |
| *Glucose (mmol/L)* | 0.0840 | 0.0465 | 3.2628 | 1.0000 | 0.0709 |
| *Potassium (mmol/L)* | 0.0854 | 0.0877 | 0.9496 | 1.0000 | 0.3298 |
| *Anemia (Yes)* | -0.0807 | 0.0870 | 0.8601 | 1.0000 | 0.3537 |
| *COPD (No)* | -0.0999 | 0.1051 | 0.9041 | 1.0000 | 0.3417 |
| *Highest Level of Education (No education)* |  |  |  |  |  |
| *Primary* | 0.1012 | 0.4111 | 0.0606 | 1.0000 | 0.8055 |
| *Lower* | 0.2659 | 0.3919 | 0.4606 | 1.0000 | 0.4974 |
| *Junior* | 0.2300 | 0.3929 | 0.3428 | 1.0000 | 0.5582 |
| *Secondary* | 0.3308 | 0.3917 | 0.7134 | 1.0000 | 0.3983 |
| *Senior* | 0.4522 | 0.4003 | 1.2757 | 1.0000 | 0.2587 |
| *Higher vocational* | 0.2936 | 0.3940 | 0.5555 | 1.0000 | 0.4561 |
| *University* | 0.4064 | 0.4083 | 0.9904 | 1.0000 | 0.3196 |
| *Other* | 0.3358 | 0.4172 | 0.6478 | 1.0000 | 0.4209 |
| *Eating Disorder (Yes)* | -0.2033 | 0.3407 | 0.3559 | 1.0000 | 0.5508 |
| *Gall Stones (Yes)* | 0.0062 | 0.1282 | 0.0024 | 1.0000 | 0.9612 |
| *Hypertension (No)* | 0.0230 | 0.0630 | 0.1340 | 1.0000 | 0.7143 |
| *I Live Alone (No)* | -0.2175 | 0.0796 | 7.4723 | 1.0000 | 0.0063 |
| *How much of the time been nervous during the past 4wks? (All the time)* |  |  |  |  |  |
| *Most of the time* | -0.1594 | 0.6851 | 0.0541 | 1.0000 | 0.8161 |
| *Often* | -0.4324 | 0.6146 | 0.4950 | 1.0000 | 0.4817 |
| *Sometimes* | -0.4019 | 0.5911 | 0.4624 | 1.0000 | 0.4965 |
| *Rarely* | -0.4671 | 0.5918 | 0.6229 | 1.0000 | 0.4300 |
| *Never* | -0.4394 | 0.5914 | 0.5520 | 1.0000 | 0.4575 |
| *How much of the time been calm & peaceful during the past 4wks? (All the time)* |  |  |  |  |  |
| *Most of the time* | -0.1108 | 0.0892 | 1.5445 | 1.0000 | 0.2139 |
| *Often* | -0.1793 | 0.1178 | 2.3159 | 1.0000 | 0.1281 |
| *Sometimes* | -0.2584 | 0.1531 | 2.8478 | 1.0000 | 0.0915 |
| *Rarely* | -0.2976 | 0.2672 | 1.2404 | 1.0000 | 0.2654 |
| *Never* | 0.0764 | 0.3191 | 0.0573 | 1.0000 | 0.8109 |
| *How much of the time been downhearted & blue past 4wks? (All the time)* |  |  |  |  |  |
| *Most of the time* | -0.0816 | 0.7627 | 0.0114 | 1.0000 | 0.9148 |
| *Often* | -0.6941 | 0.7031 | 0.9746 | 1.0000 | 0.3235 |
| *Sometimes* | -0.4729 | 0.6901 | 0.4696 | 1.0000 | 0.4932 |
| *Rarely* | -0.5771 | 0.6905 | 0.6983 | 1.0000 | 0.4033 |
| *Never* | -0.5863 | 0.6901 | 0.7218 | 1.0000 | 0.3956 |
| *How much of the time had a lot of energy during the past 4wks? (All the time)* |  |  |  |  |  |
| *Most of the time* | 0.1572 | 0.1129 | 1.9407 | 1.0000 | 0.1636 |
| *Often* | 0.0947 | 0.1295 | 0.5348 | 1.0000 | 0.4646 |
| *Sometimes* | 0.0284 | 0.1473 | 0.0372 | 1.0000 | 0.8471 |
| *Rarely* | 0.2433 | 0.2049 | 1.4104 | 1.0000 | 0.2350 |
| *Never* | 0.0768 | 0.3483 | 0.0486 | 1.0000 | 0.8255 |
| *How much of the time feeling worn out during the past 4wks? (All the time)* |  |  |  |  |  |
| *Most of the time* | 0.4971 | 0.5380 | 0.8538 | 1.0000 | 0.3555 |
| *Often* | 0.1597 | 0.5097 | 0.0982 | 1.0000 | 0.7540 |
| *Sometimes* | 0.0964 | 0.5100 | 0.0357 | 1.0000 | 0.8501 |
| *Rarely* | 0.0467 | 0.5124 | 0.0083 | 1.0000 | 0.9273 |
| *Never* | 0.0545 | 0.5135 | 0.0113 | 1.0000 | 0.9155 |
| *How much of the time feeling tired during the past 4wks? (All the time)* |  |  |  |  |  |
| *Most of the time* | -0.2342 | 0.2807 | 0.6964 | 1.0000 | 0.4040 |
| *Often* | -0.0670 | 0.2594 | 0.0667 | 1.0000 | 0.7962 |
| *Sometimes* | 0.2557 | 0.2666 | 0.9200 | 1.0000 | 0.3375 |
| *Rarely* | 0.2764 | 0.2740 | 1.0177 | 1.0000 | 0.3131 |
| *Never* | 0.4042 | 0.2856 | 2.0034 | 1.0000 | 0.1570 |
| *How would you rate your health, generally speaking? (Excellent)* |  |  |  |  |  |
| *Very good* | -0.0905 | 0.1053 | 0.7384 | 1.0000 | 0.3902 |
| *Good* | 0.0376 | 0.1099 | 0.1173 | 1.0000 | 0.7320 |
| *Mediocre* | 0.2163 | 0.1626 | 1.7703 | 1.0000 | 0.1834 |
| *Poor* | 0.8187 | 0.5059 | 2.6190 | 1.0000 | 0.1056 |
| *Physical health limited work during past 4wks? (Yes)* | -0.0422 | 0.0961 | 0.1931 | 1.0000 | 0.6603 |
| *I expect my health to get worse (Completely false)* |  |  |  |  |  |
| *Mainly false* | -0.0230 | 0.0903 | 0.0647 | 1.0000 | 0.7993 |
| *I do not know* | 0.0491 | 0.0702 | 0.4887 | 1.0000 | 0.4845 |
| *Mainly true* | 0.0024 | 0.1034 | 0.0005 | 1.0000 | 0.9816 |
| *Completely true* | 0.0697 | 0.1914 | 0.1328 | 1.0000 | 0.7155 |
| *My health is excellent (Completely false)* |  |  |  |  |  |
| *Mainly false* | 0.1588 | 0.1998 | 0.6318 | 1.0000 | 0.4267 |
| *I do not know* | 0.1505 | 0.1937 | 0.6040 | 1.0000 | 0.4370 |
| *Mainly true* | 0.0354 | 0.1820 | 0.0379 | 1.0000 | 0.8457 |
| *Completely true* | 0.1059 | 0.1861 | 0.3241 | 1.0000 | 0.5691 |
| *Sleep (4 hours)* |  |  |  |  |  |
| *5 hours* | -0.0065 | 0.4813 | 0.0002 | 1.0000 | 0.9892 |
| *6 hours* | -0.0305 | 0.4218 | 0.0052 | 1.0000 | 0.9423 |
| *7 hours* | 0.1120 | 0.4184 | 0.0717 | 1.0000 | 0.7889 |
| *8 hours* | 0.0897 | 0.4170 | 0.0463 | 1.0000 | 0.8297 |
| *9 hours* | 0.3304 | 0.4278 | 0.5965 | 1.0000 | 0.4399 |
| *10 hours* | 0.5150 | 0.4699 | 1.2010 | 1.0000 | 0.2731 |

**Hazard Ratio Plots of Other Important Variables**
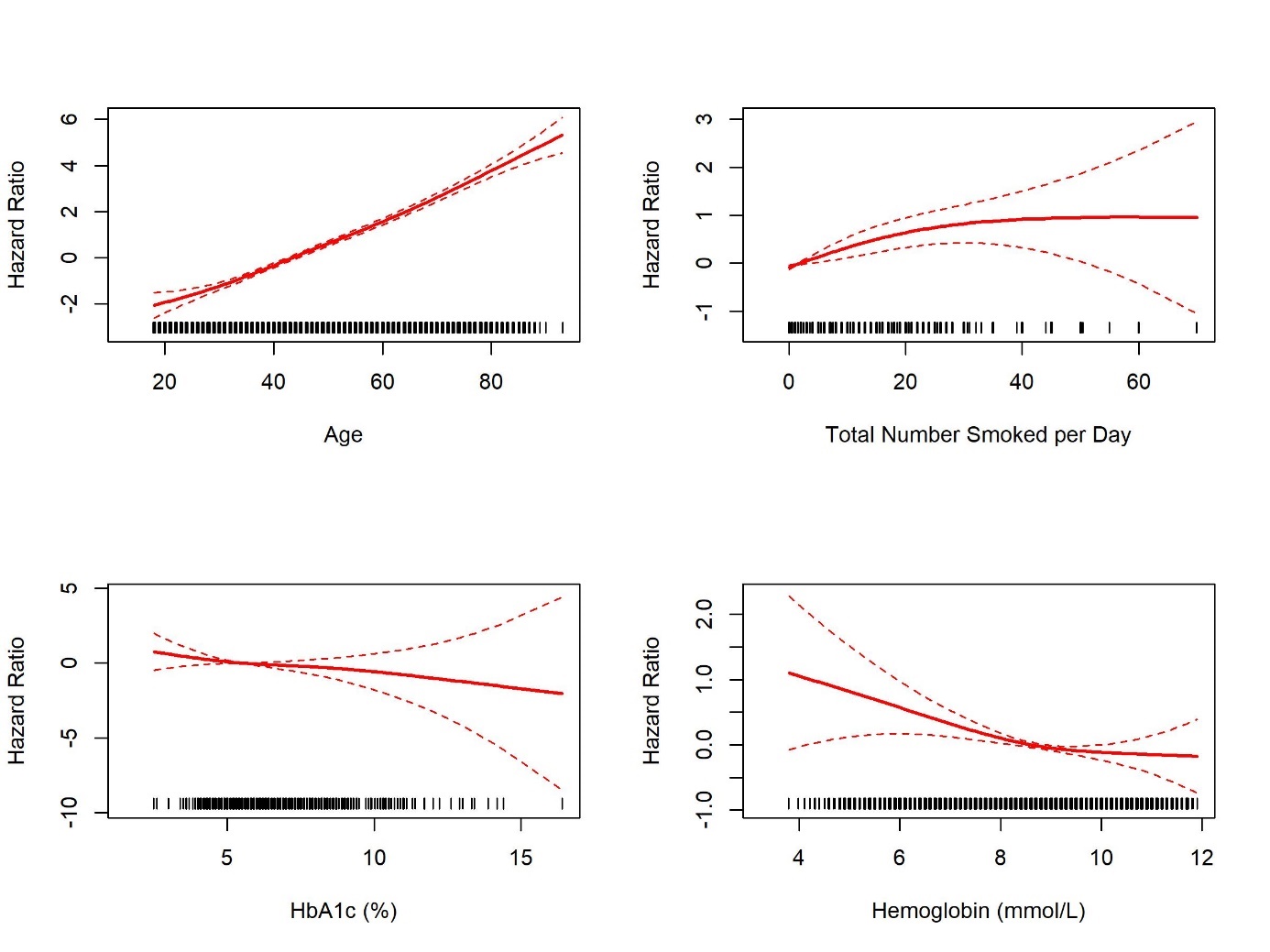

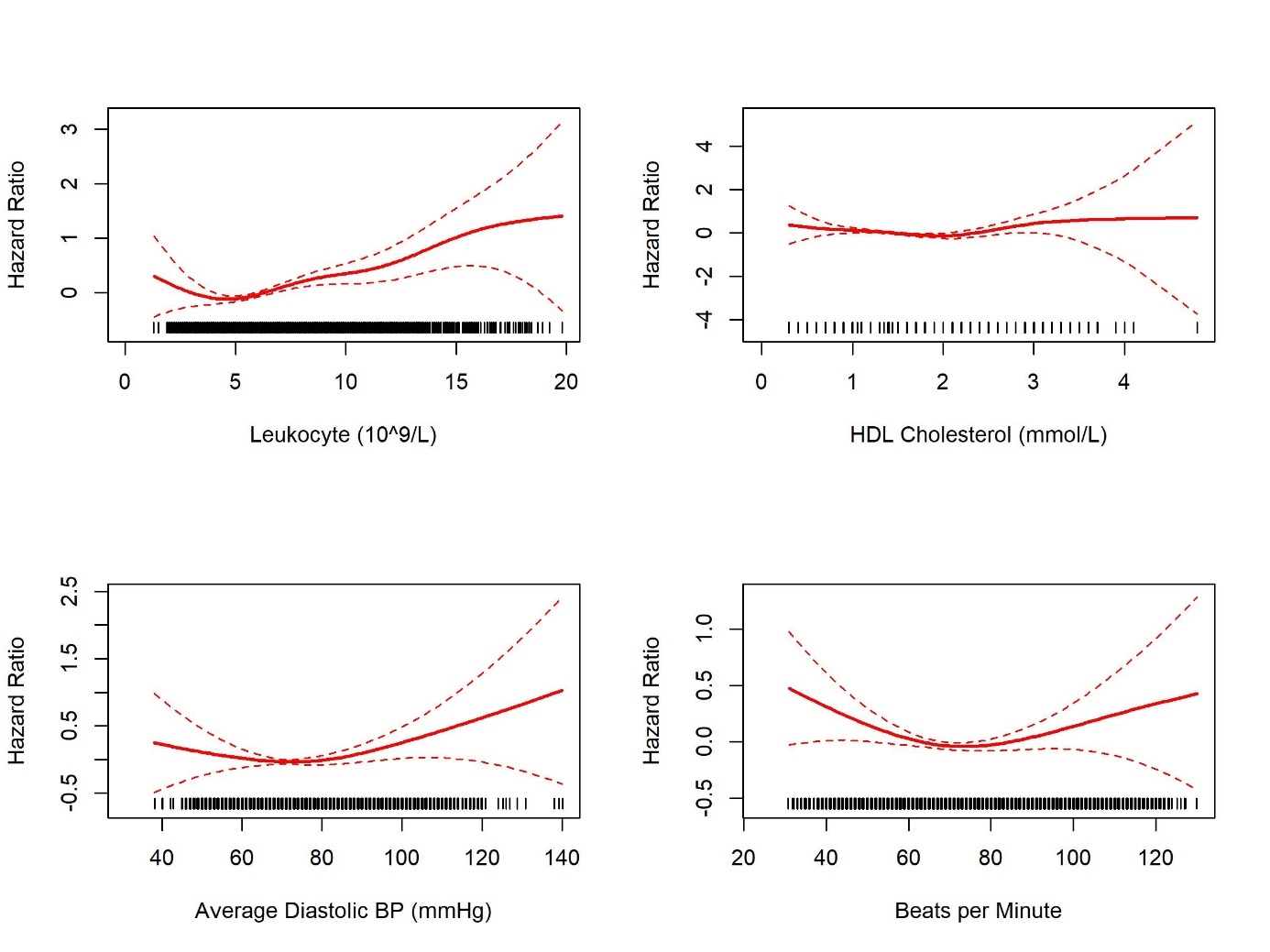

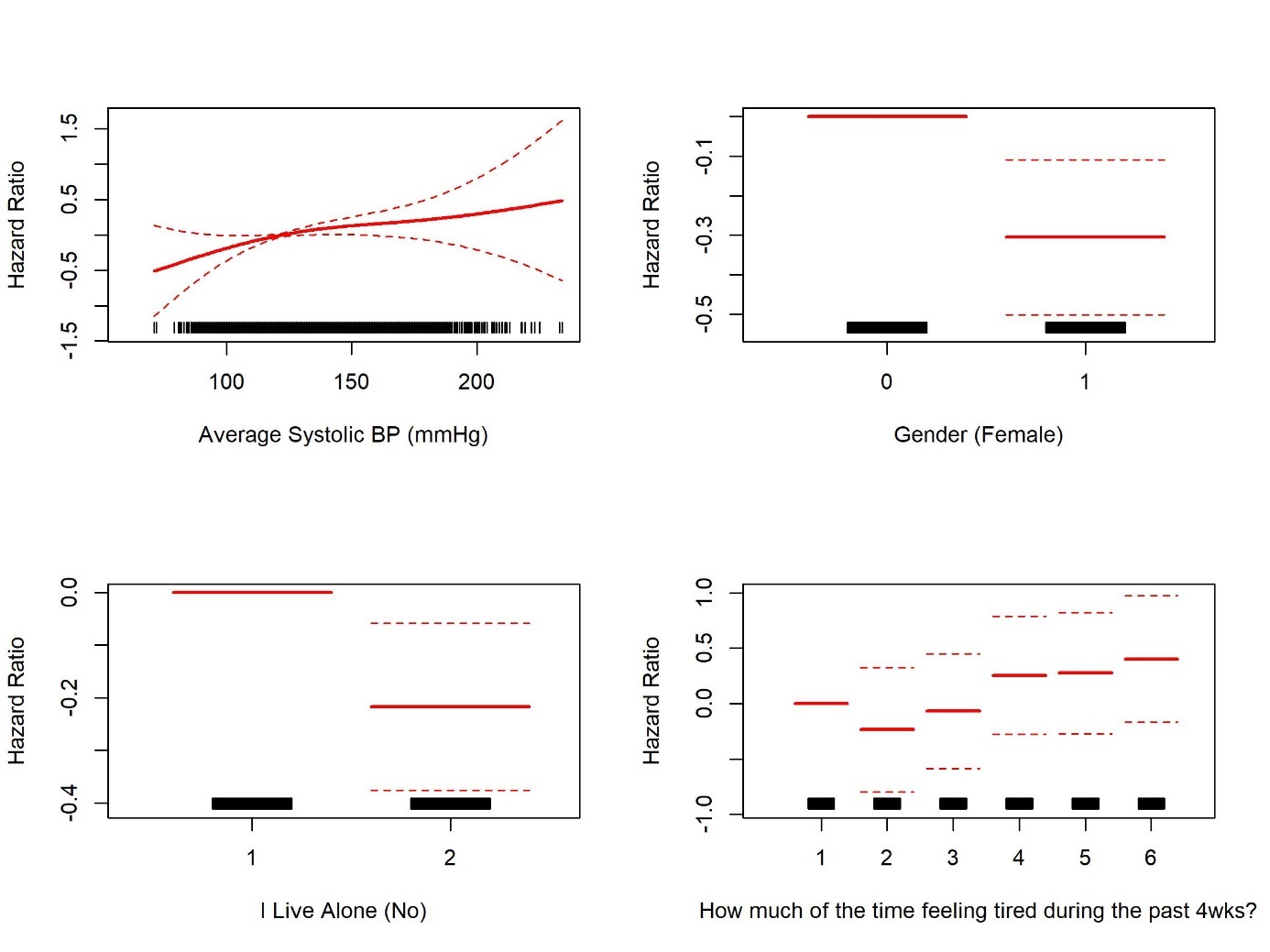


Figure S4: HR plots of the most important variables identified by likelihood ratio tests.

**“How much time feeling tired during the past 4 weeks” age characteristics**


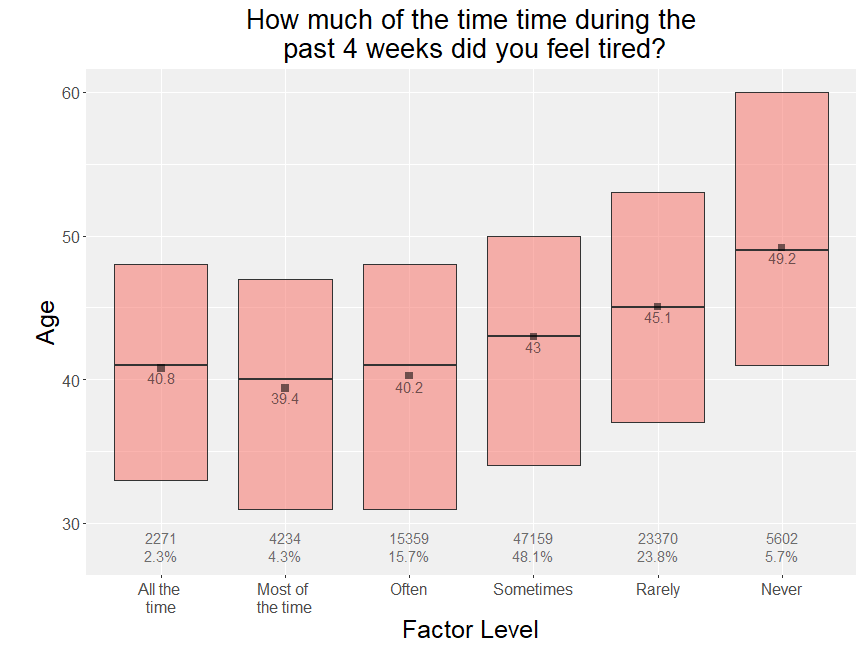


Figure S5: The absolute number and percentage of people (above the factor level labels along the x-axis) responding to each level of the question “How much time feeling tired during the past 4 weeks” and their corresponding age characteristics is shown. The black lean represents the median age and the square the mean age.
